# Supplementary material for: StripePy: fast and robust characterization of architectural stripes
Source: Bioinformatics. 2025 Jun 13;41(6):btaf351. doi: 10.1093/bioinformatics/btaf351 (PMC12215313; doi:10.1093/bioinformatics/btaf351)
Supplement: btaf351_Supplementary_Data [file btaf351_supplementary_data.pdf]

Supplementary Data

StripePy: fast and robust characterization of architectural stripes

Andrea Raffo[1], Roberto Rossini[1], and Jonas Paulsen[1]

[1]Department of Biosciences, University of Oslo, 0316, Oslo, Norway

Extended description of StripePy

StripePy runs through four consecutive steps: a pre-processing (Step 1), followed by the identification of genomic bins exhibiting linear patterns (Step 2), the subsequent estimation of geometric descriptors (Step 3), and the final definition of biological descriptors (Step 4). A compact overview of the algorithm is given in Fig. 1, while a more detailed representation is provided in Supplementary Fig. 7.

Step 1: Laying the groundwork for data exploration

To mitigate the bias of the *genomic distance effect* – namely the tendency of higher prevalence of crosslinks between genomic loci close together along the genome even in the absence of any specific higher order structure – we perform global enhancement via the well-known log-transformation. The log-transformation is a global intensity transformation which expands narrow ranges of low intensity values and compresses wide ranges of high intensity values. The general form of the log-transformation is

$$P(x, y) = c \log(1 + H(x, y)), \tag{1}$$

being $H(x, y) \geq 0$ the initial intensity at bin pair (x, y), $P(x, y)$ the output intensity at the same location, and c a constant. Here, we set c so that intensities are projected onto $[0, 1]$. Furthermore, we retain only entries that fall within a specific band around the main diagonal (default bandwidth: 5 Mbp), as we are specifically targeting patterns anchored at the main diagonal. As an example, Supplementary Fig. 7A-B provides a graphical illustration of how global enhancement acts on a specific square sub-region along the

main diagonal (what we will call a *region of interest*).

We proceed by splitting the pre-processed Hi-C matrix P into lower- and upper-triangular matrices, henceforth denoted as L and U, respectively; see Supplementary Fig. 7C-D.

Step 2: Line detection via pattern recognition and Topological Data Analysis

For the sake of conciseness, we here adopt two simplifications. First and foremost, we restrict our explanation on the lower-triangular matrix, i.e., L – as the processing of the upper-triangular part U is analogous; for visualization purposes, however, we will often show the full Hi-C matrix P. We choose to display the full matrix as it can help the visual analysis of an algorithm's output, as any vertical pattern can be found horizontally given that Hi-C matrices are naturally symmetric. Secondly, we limit ourselves to recognizing vertical patterns, again because of symmetry.

We sum L over the rows, thus obtaining a scalar function, or 1D profile, which can be interpreted as a *pseudo-distribution* π_{glob}. The function is smoothed by using weighted quasi-interpolation [Raffo and Biasotti(2021a), Raffo and Biasotti(2020), Raffo and Biasotti(2021b)], a weighted moving average technique for curve approximation. A graphical example is given in Supplementary Fig. 7E. The term "pseudo" used in this context is necessary as π_{glob} is not, strictly speaking, a probability distribution. The location of each local maximum points at a possible linear pattern. Note that the pseudo-distribution bears resemblance to the accumulator function derived by applying the Hough Transform [Hough(1962), Mukhopadhyay and Chaudhuri(2015)], but in this case the search for vertical linear patterns is conducted without any edge detection or binarization to speed up computation and avoid the introduction of additional parameters.

We filter local maxima by measuring their significance via *topological persistence*. In the analogy of the water level, the pseudo-distribution is a submerged bank. Imagine the water level to gradually go down: when it reaches a local maximum, a new island is formed, and its height is called *birth level*; when it reaches a local minimum, two islands merge. The higher island is, by convention, considered to subsume the lower island, and the local minimum is the lower island's *death level*. The topological persistence of an island, i.e., its significance, is the difference between its birth and death levels. The local maximum points of π_{glob} that survive the filtration process will be here referred to as the *seed sites* of candidate stripes, and will be denoted as $s_1 < s_2 < \cdots < s_n$. An example of seed sites is provided in Supplementary Fig. 7F, while the corresponding locations are superimposed to the contact map in Supplementary Fig. 7G.

To avoid spurious seed sites from sparse regions, we apply the following additional check. For each seed site, we consider a neighborhood of pre-defined size and calculate the ratio of bins – within this neighborhood – where π_{glob} exceeds 0.1 (i.e., 10% of the maximum height); if this ratio is greater than 85%, the seed site

is retained, otherwise it is discarded.

Step 3: Shape analysis

Once the seed sites (i.e., sites hosting linear patterns) $s_1, \ldots, s_n$ are detected in the lower-triangular pre-processed Hi-C matrix L, we proceed by estimating width and height of each candidate stripe S_j independently as follows.

Width estimation We start by defining a sub-interval $I_j := [i_j^L, i_j^R] \subset I$ so that I_j is the largest interval with the following properties: (1) s_j is contained in I_j; (2) π_{glob} is monotonically increasing in $[i_j^L, s_j]$; (3) π_{glob} is monotonically decreasing in $[s_j, i_j^R]$. To define the width of of S_j, we look at the global pseudo-distribution π_{glob} in the interval I_j. The right boundary of the candidate stripe is defined as

$$b^R(s_j) := \min\{\tilde{b}^R(s_j), b + w_{\max}/2\}, \tag{2}$$

where

$$\tilde{b}^R(s_j) := \arg\max_b \left\{ \pi_{\text{glob}}(b) - \pi_{\text{glob}}(b-1) \middle| s_j < b \leq i_j^R \right\}; \tag{3}$$

the left boundary is given by

$$b^L(s_j) := \max\{\tilde{b}^L(s_j), b - w_{\max}/2\}, \tag{4}$$

where

$$\tilde{b}^L(s_j) := \arg\max_b \left\{ \pi_{\text{glob}}(b+1) - \pi_{\text{glob}}(b) \middle| i_j^L \leq b < s_j \right\}. \tag{5}$$

Here, $w_{\max}$ denotes the maximum width, which by default is set to a value of 100 kb.

These formulas correspond to defining the boundaries as the genomic bins exhibiting largest values of (approximate) first-order derivative of π_{glob} in a left or right neighbourhood of s_j, with a maximum width as additional constraint. An exemplification of this description is shown in Supplementary Fig. 7H. Given a seed site, the left and right boundaries naturally define horizontal intervals, sometimes referred to as horizontal domains: see Supplementary Fig. 7I for a graphical display of what the single horizontal domains mean in terms of (lower-triangular) matrix slicing.

The width of a candidate stripe is computed by $b^R(s_j) - b^L(s_j)$.

Height estimation Given the j-th seed site and the corresponding horizontal domain made of left and right boundaries, we define a vertical domain (lower and upper boundaries) by introducing a *local* pseudo-

distribution. Such a profile is obtained by applying the following procedure (also see Supplementary Fig. 7I-J):

- Slice L: filter out columns that do not belong to the current horizontal domain; discard rows from the upper-triangular part of the matrix.
- Sum (i.e., marginalize) over the surviving columns, then scale by dividing by the global maximum. Smooth the local pseudo-distribution by using, again, the weighted quasi-interpolation.

Two criteria are available: (1) By applying topological persistence to find persistent maxima, we can determine where the local 1D pseudo-distribution presents significant peaks; we use the peak that is farthest from the main diagonal to define the stripe boundary. (2) The vertical domain of a candidate stripe is retrieved by finding the smallest interval containing all coordinates where the smooth approximation is above a user-threshold. When studying a candidate stripe in the lower-triangular matrix, the top-boundary $b^U(s_j)$ is naturally anchored to the main diagonal; for bottom-boundary $b^D(s_j)$, the steepest the local pseudo-distribution, the closer $b^D(s_j)$ is to the main diagonal. The height of a stripe is then computed as $b^D(s_j) - b^U(s_j)$.

At this point, each quintuplet consisting of seed site, left-, right-, top-, and bottom-boundaries, defines uniquely a candidate stripe from a geometric viewpoint. We will denote candidate stripes as $S_1, \dots, S_n$ to distinguish them from seed sites $s_1, \dots, s_n$; an example is shown in Supplementary Fig. 7K.

Step 4: Signal analysis

To enrich a purely geometric structure with biological information, we compute descriptors that summarize information about the signal distribution – both inside and outside of candidate stripes. To prevent our descriptors from being influenced by the genomic distance effect, we apply a correction to the shape of a stripe. Instead of selecting a number of rows equal to the stripe's height, we select a number of diagonals matching its height, resulting in a parallelogram, see Supplementary Fig. 7L.

- *Inner descriptors* We adopt the five-number summary, a set of descriptive statistics consisting of five sample percentiles:
  - The minimum, sometimes called Q_0 or 0th percentile.
  - First (Q_1), second (Q_2), and third (Q_3) quartiles – which consists of the 25th, 50th, and 75th percentile, respectively. The second quartile corresponds to the median; the first and third quartiles correspond to the median of the lower and the upper half of the dataset, respectively.

- The maximum, sometimes called Q_4 or 100th percentile.

In addition, we consider the arithmetic mean and standard deviation of the signal within the stripe boundaries. We will call the latter as *mean inner signal*.

- *Outer descriptors.* We compute the statistics from the previous point using regions that neighbor the candidate stripe. More precisely, the *left k-neighbour* $\mathcal{N}_j^R$ consists of the first k diagonals to the left of S_j; analogously, the *right k-neighbour* $\mathcal{N}_j^R$ consists of the first k diagonals to the right of S_j. We will denote as $\mathcal{N}_j := \mathcal{N}_j^L \cup \mathcal{N}_j^R$ the *k-neighbour* of the j-th candidate stripe. The arithmetic mean of the signal inside the k-outer neighbour will be referred to as *mean k-outer signal*.

- *Mixed descriptors.* We define the *k-relative change* as the difference between mean inner and mean k-outer signals, divided by the mean k-outer signal. The relative change resembles the estimation of a first-order derivative at the stripe boundary, when performed by using a first-order finite difference method.

Extended description of StripeBench

This section is devoted to provide additional information on the StripeBench benchmark, intended as a dataset equipped of a ground truth and a set of performance measures.

Dataset

To generate Hi-C matrices we consider a genome-wide run of MoDLE with loop extrusion simulated by using barriers imputed from RAD21 ChIP-Seq H1-hESC data, and with default settings except for the following three factors:

- Target contact density δ, i.e., the average contact density to be reached before the simulation is halted, here set to 1, 5, 10, and 15.

- Scale parameter σ of the generalized extreme value distribution, used to randomize contacts by additively introducing noise to molecular contacts, is set to 0, 5000, 10000, and 15000.

- Resolution r in base-pairs for the output contact matrix in cooler format, set to 5k, 10k, 25k and 50k.

The values chosen for the three factors lead to 16 Hi-C matrices, each one available at four resolutions for a total of 64 simulated matrices. The choice of target contact density and scale parameters in our study is driven by the objective of considering Hi-C matrices with diverse sequencing depths and increasing levels

of noise, while still maintaining a manageable number of matrices. For a graphical illustration of the effects of these parameters on the contact maps, the reader is referred to Fig. 2A.

Ground truth

For each Hi-C matrix, the ground truth is obtained by retrieving loci with average extrusion barrier occupancy greater or equal than 0.70. Extrusion barrier occupancy consists of a scalar in $[0, 1]$, and is therefore interpretable as a probability value; intuitively, the higher this value, the more visible the stripe is, as shown in visual examples in Fig. 2B.

We then proceed by merging extrusion barriers mapping to the same bin at a given resolution – so that each locus appears only once; however, we consider the lower- and the upper-triangular parts of the contact map separately, so that a bin can yet be deemed relevant for both parts of the matrix, either individual parts or neither of them. Each of loci will be henceforth referred to as *anchor site* or *anchor point*.

Different measures for different problems

The capability of a tool to find meaningful stripes is here quantified by using classification and recognition measures. We hereby adopt the following terminology:

- *Classification* is the task of assigning labels to input data, such as categorizing genomic bins as either 0 or 1 based on whether or not they contain an anchor point. We therefore discard the notion of width and evaluate a method's ability to identify whether a certain bin *hosts (i.e., contains)* an anchor point or not. For Chromosight and StripeCaller, obtaining the predicted classification vector is straightforward since these methods specifically set the stripe width to the resolution of the Hi-C matrix[1]; in the case of StripePy, we obtain the predicted classification vector by considering the coordinates of seed points as anchor point predictions; for Stripenn we consider the midpoint of each horizontal interval, since the method does not provide additional information to help picking a location.

- *Recognition* deals with the identification of rectangular regions[2] that host anchor points.

We decide to employ both formulations since neither of them alone is adequate for analysing the problem. Solely relying on recognition is insufficient to assess a set of tools because any partition of the Hi-C matrix can capture all anchor sites with no difficulty, albeit with a potentially excessive number of wide stripes. This limitation originates because it is not possible to obtain a ground truth notion of width or length. On

[1] These two methods return pairs of the form $\{i, i + r\}$, where r is the resolution and i is a genomic coordinate. We consider the bin of genomic coordinate i to host the anchor point.

[2] What stripes are, geometrically speaking

the other hand, classification measures does not account that stripes are not unidimensional segments, but rather narrow rectangles.

The following "base" measures are adopted for the classification problem:

- *Sensitivity and Specificity* measure the ability of a method to correctly identify positives and negative cases, respectively. By *positives* we here mean bins that are flagged as anchor points, while *negatives* are bins that do not host any anchor. Sensitivity is also known as *True Positive Rate* (TPR) or *recall*, while specificity goes under the name of *True Negative Rate* (TNR).

- *Positive Predictive Value* (PPV), or *precision*, is the proportion of true positives to the total number of items classified as positives by the model.

Similarly, we introduce the following "base" recognition measures:

- *Anchor Hit Rate*, or AHR, computed as the fraction of anchor sites that lie in one of the candidate stripes. It is closely related to TPR, although we are here more permissive since we consider the estimated stripe width in its computation.

- *Fraction of Good Candidates*, or FGC, computed as the fraction predicted stripes hosting an anchor site. It resembles to PPV when accounting for the estimated stripe width.

It is worth noting that there is no metric corresponding to specificity, as we are only able to draw conclusions on candidate stripes (whether these are true or false positives) since we lack a notion of negative.

There are several metrics that are used to handle datasets with a severe skew in the class distribution. The following single-value metrics are adopted:

- *Balanced accuracy* and *Geometric Mean* are single-value metrics that combine sensitivity and specificity by computing their arithmetic or geometric mean. We denote them as bACC, and GM, respectively.

- *F1-score* and *Fowlkes–Mallows index*, here respectively denoted as F1c and FMc, are the harmonic and geometric means of precision and recall.

- *Jaccard Index* (JI), or Jaccard similarity coefficient, measures the similarity between true labels and predicted labels by taking the ratio between the number of true positives against the sum of correctly predicted positive samples and all incorrect predictions. This metric considers both false positives and false negatives in a balanced manner, excluding only the correctly predicted negative samples. It is particularly well-suited for detecting rare events, as it ensures that the evaluation is sensitive to the correct classification of infrequent positive instances without being dominated by numerous correct identifications of negative class instances.

In a similar manner, recognition measures are summarized by adapting F1-score and Fowlkes–Mallows index: in this adaptation, TPR is replaced by AHR and PPV is replaced by FGC. We denote them as F1r and FMr, respectively.

Benchmarking analysis

The following tools were installed for the comparative analyses: Chromosight version 1.6.3 [Matthey-Doret et al.(2020)Matthey-Doret, Baudry, Breuer, and et al.], StripeCaller version 0.1.0 `https://github.com/XiaoTaoWang/StripeCaller`, Stripenn 1.1.65.18 [Sora Yoon et al.(2022)Sora Yoon, Aditi Chandra, and Vahedi], and StripePy version 1.0.0.

Matrix normalization affects StripePy performance

We decided to investigate the effect of contact map normalization on the performance of StripePy. To this end, maps from Supplementary Table 9 were normalized using hictk [Rossini and Paulsen(2024)] with two algorithms: genome-wide iterative correction and eigenvector decomposition (ICE) [Imakaev et al.(2012)Imakaev, Fudenberg, McCord, Naumova, Goloborodko, Lajoie, Dekker, and Mirny] and genome-wide SCALE [Robinson et al.(2018)Robinson, Turner, Durand, Thorvaldsdóttir, Mesirov, and Aiden]. To ensure a consistent comparison, StripePy was rerun on the normalized contact maps with all thresholds unchanged. Snapshots of neighborhoods are provided in Supplementary Fig. 6, while classification and recognition measures are reported in Supplementary Table 9.

We first note that applying normalization significantly reduces the number predicted stripes (nSP). This reduction is visually evident when comparing the plots for the case of no normalization (Supplementary Fig. 6 A1, B1, C1, and D1) with those for ICE- (Supplementary Fig. 6 A2, B2, C2, and D2) and SCALE-normalized maps (Supplementary Fig. 6 A3, B3, C3, and D3). This observation is further confirmed by the nSP values in Supplementary Table 9: for example, for the H1-hESC Micro-C map, nSP decreases from 26,210 to 6,303 and 8,819 for raw, and ICE- and SCALE-normalized maps, respectively. This is not surprising, as one of the effects of Hi-C matrix normalization is smoothing of the original data, resulting in smoothed global pseudo-distributions and, consequently, a smaller number of peaks (i.e., stripe seeds).

Other interesting observations can be made from Supplementary Table 9. First, the decrease in nSP is accompanied by a decrease in the number of found anchors (nAF): for the same map, nAF decreases from 32,644 to 14,722 and 18,708 with ICE- and SCALE-normalized maps, respectively. Moving to the analysis of classification and recognition measures, we observe that for all contact maps, applying normalization

increases some base metrics (TNR and FGC) while decreasing others (TPR, PPV and AHR). By examining single-value metrics, it becomes evident that the gain in the former scores is not sufficient to compensate the drop in the latter metrics, resulting in a unanimous decrease in all single-value statistics. Therefore, we conclude that normalizing contact maps before using StripePy for stripe calling might impair performance and should be approached with caution.

Impact of the two filtering strategies

StripePy employs two filtering strategies. The first filtration takes place in Step 2 (see Methods), where local maximum points of the global pseudo-distribution are filtered by thresholding their topological persistence values. By applying this filtration to the global pseudo-distribution, we retain genomic bins with a stronger (uni-dimensional) linear pattern, as this process resemble the voting procedure from the Hough transform [Hough(1962)]. The effect of increasing thresholds on these local maximum points is illustrated in Supplementary Fig. 8A-C: in practice, requiring higher topological persistence prioritizes local maxima with greater height differences compared to their surrounding areas. Supplementary Fig. 8 D-E show the candidate stripes produced at the end of Step 3 (see Methods), where each seed site is enriched with estimates of stripe width and height. While acting on topological persistence already reduces the number of candidate stripes, it doesn't account for stripes being two-dimensional patterns. Therefore, a second filtering step is applied, which thresholds the relative change parameter – a score that compares the average signal inside and in the near proximity of a candidate stripe. The effect of a more stringent filtering on this parameter for the set of candidates of Supplementary Fig. 8E is displayed in Supplementary Fig. 8 G-I. As expected, the higher the threshold, the fewer candidates are returned.

Default values for these thresholds are provided with the software to simplify the user's work and enable a fair comparison with alternative stripe callers. However, optimal results may require adjusting these parameters based on the specific contact map. It is often best to run the tool on a chromosome and fine-tune the parameter values on a case-by-case basis to achieve the most accurate results.

Pre-processing of interaction matrices released by ENCODE and the 4DNucleome

Matrices in .mcool format published by the 4DNucleome are generated using a version of Cooler that leads to data corruption when generating the .mcool files by iterative coarsening a .cool file [3]. Thus, prior to using dataset 4DNFI6HDY7WZ and 4DNFI9GMP2J8 [Nucleome(a), Nucleome(b)] for any data analysis or benchmark, we restored the corrupted files using `hictk fix-mcool` [Rossini and Paulsen(2024)].

Next, interaction matrices in .hic format were converted to .mcool format using `hictk convert` [Rossini and Paulsen(2024)].

References

[Raffo and Biasotti(2021a)] Andrea Raffo and Silvia Biasotti. Weighted quasi-interpolant spline approximations: Properties and applications. *Numerical Algorithms*, 87(2):819–847, 2021a. DOI:10.1007/s11075-020-00989-4.

[Raffo and Biasotti(2020)] Andrea Raffo and Silvia Biasotti. Data-driven quasi-interpolant spline surfaces for point cloud approximation. *Computers & Graphics*, 89:144–155, 2020. DOI:10.1016/j.cag.2020.05.004.

[Raffo and Biasotti(2021b)] Andrea Raffo and Silvia Biasotti. Weighted Quasi-Interpolant Spline Approximations of Planar Curvilinear Profiles in Digital Images. *Mathematics*, 9(23), 2021b. DOI:10.3390/math9233084.

[Hough(1962)] P. V. C. Hough. Method and means for recognizing complex patterns. US Patent 3069654, 12 1962.

[Mukhopadhyay and Chaudhuri(2015)] Priyanka Mukhopadhyay and Bidyut B. Chaudhuri. A survey of Hough Transform. *Pattern Recognition*, 48(3):993–1010, 2015. DOI:10.1016/j.patcog.2014.08.027.

[Matthey-Doret et al.(2020)Matthey-Doret, Baudry, Breuer, and et al.] Cyril Matthey-Doret, Lyam Baudry, Axel Breuer, and et al. Computer vision for pattern detection in chromosome contact maps. *Nature Communications*, 11(1):5795–5795, 2020. DOI:10.1038/s41467-020-19562-7.

[3] Cooler data corruption: https://hictk.readthedocs.io/en/stable/file_validation.html#cooler-index-corruption

[Sora Yoon et al.(2022)Sora Yoon, Aditi Chandra, and Vahedi] Sora Yoon, Aditi Chandra, and Golnaz Vahedi. Stripenn detects architectural stripes from chromatin conformation data using computer vision. *Nature Communications*, 13(1), 2022. DOI:10.1038/s41467-022-29258-9.

[Rossini and Paulsen(2024)] Roberto Rossini and Jonas Paulsen. hictk: blazing fast toolkit to work with .hic and .cool files. *Bioinformatics*, 40(7):btae408, 2024. DOI:10.1093/bioinformatics/btae408.

[Imakaev et al.(2012)Imakaev, Fudenberg, McCord, Naumova, Goloborodko, Lajoie, Dekker, and Mirny] Maxim Imakaev, Geoffrey Fudenberg, Rachel Patton McCord, Natalia Naumova, Anton Goloborodko, Bryan R Lajoie, Job Dekker, and Leonid A Mirny. Iterative correction of Hi-C data reveals hallmarks of chromosome organization. *Nature Methods*, 9(10):999–1003, 2012. DOI:10.1038/nmeth.2148.

[Robinson et al.(2018)Robinson, Turner, Durand, Thorvaldsdóttir, Mesirov, and Aiden] James T. Robinson, Douglass Turner, Neva C. Durand, Helga Thorvaldsdóttir, Jill P. Mesirov, and Erez Lieberman Aiden. Juicebox.js Provides a Cloud-Based Visualization System for Hi-C Data. *Cell Systems*, 6(2):256–258.e1, 2018. DOI:10.1016/j.cels.2018.01.001.

[Nucleome(a)] 4D Nucleome. 4DNFI6HDY7WZ.mcool; 4DN Data Portal — identifiers.org. https://identifiers.org/4dn:4DNFI6HDY7WZ, a. [Accessed 06-12-2024].

[Nucleome(b)] 4D Nucleome. 4DNFI9GMP2J8.mcool; 4DN Data Portal — identifiers.org. https://identifiers.org/4dn:4DNFI9GMP2J8, b. [Accessed 06-12-2024].

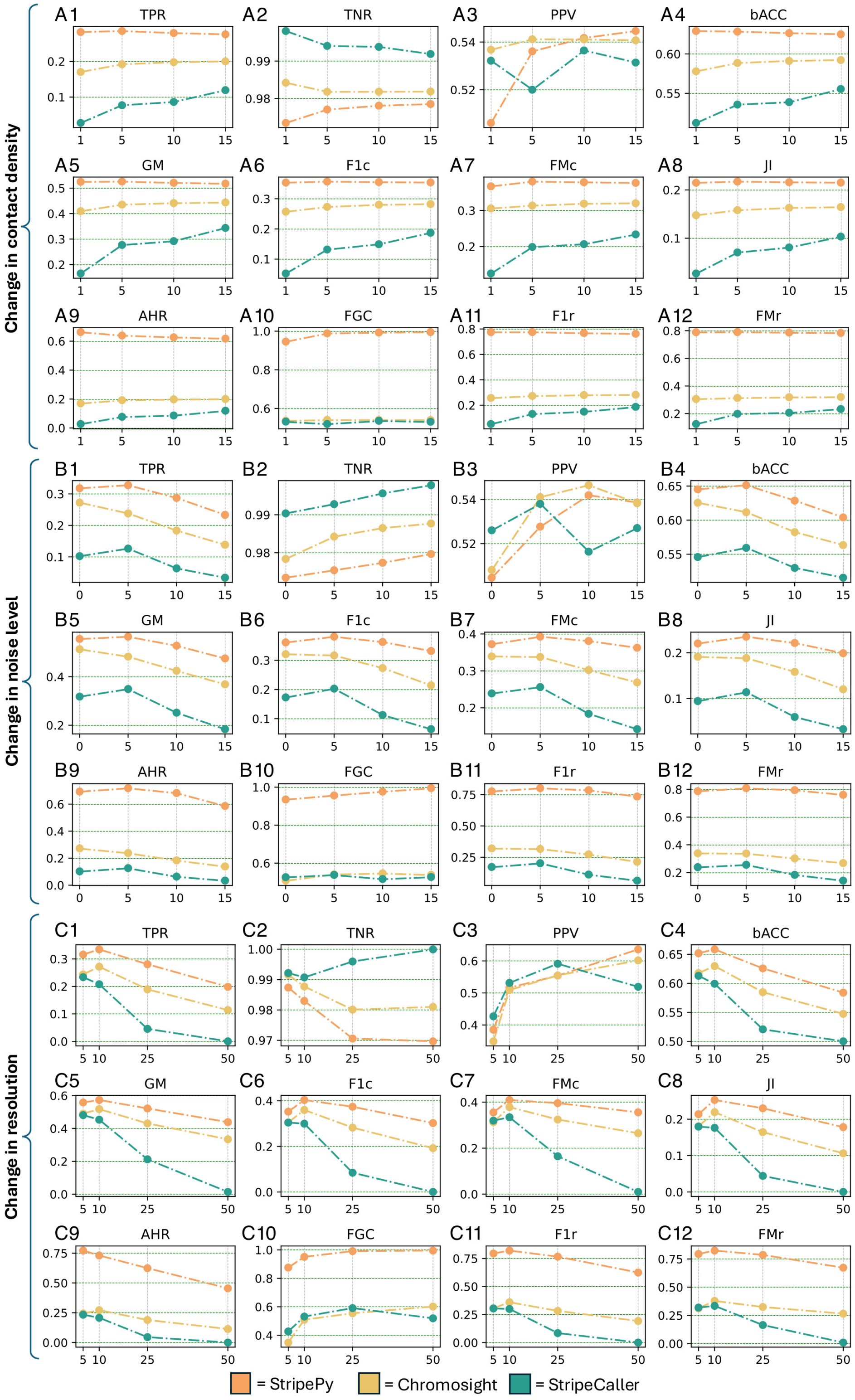

Supplementary Fig. 1. | Benchmarking pt.2. Plots of the medians of the classification and recognition measures by single factors: contact density (**A1-A12**), noise level (**B1-B12**), and resolution (**C1-C12**).

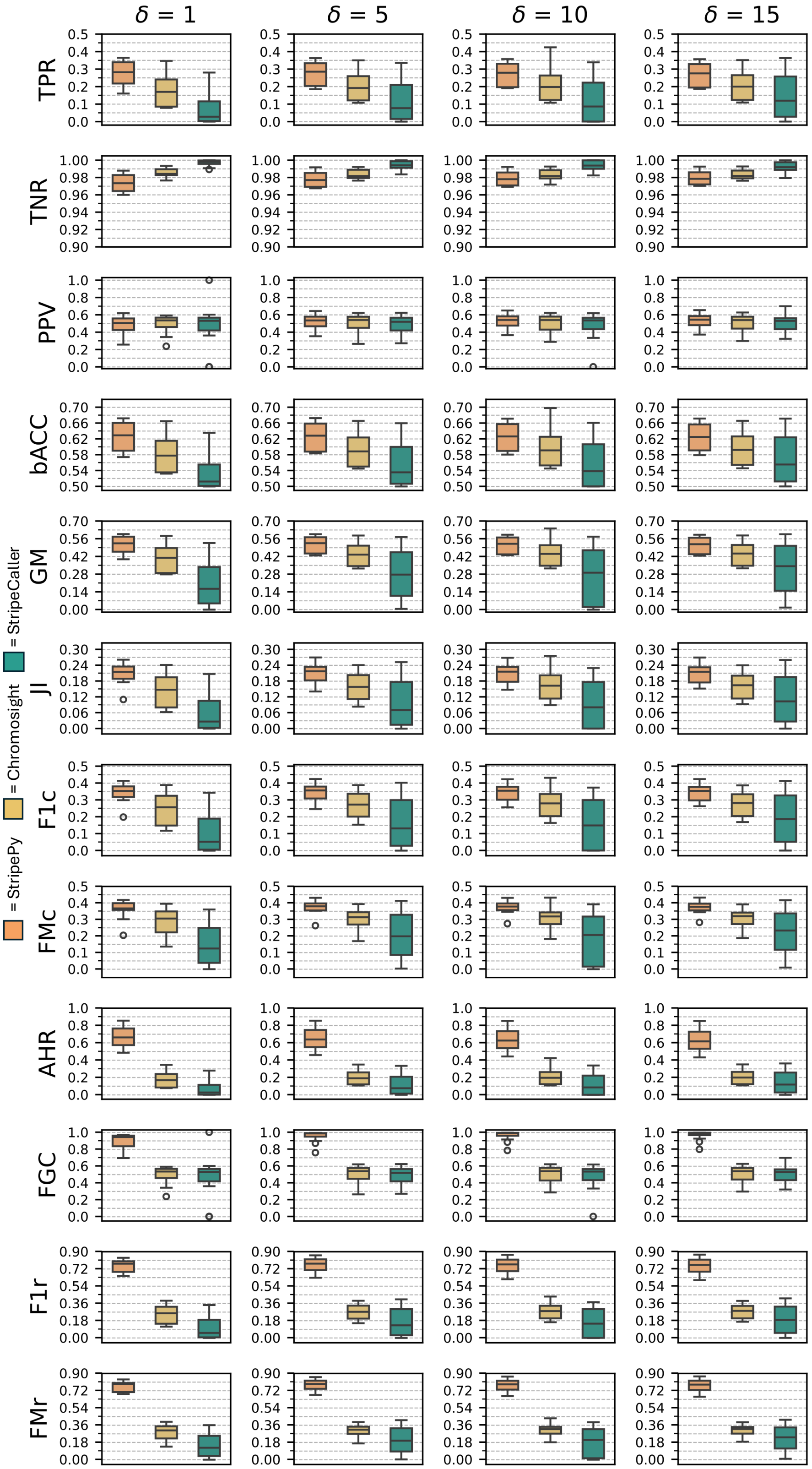

Supplementary Fig. 2. | Change in contact density for the StripeBench's benchmark. Boxplots of the classification (TPR, TNR, PPV, bACC, GM, JI, F1c, and FMc) and recognition (AHR, FGC, F1r, and FMr) measures by change in contact density.

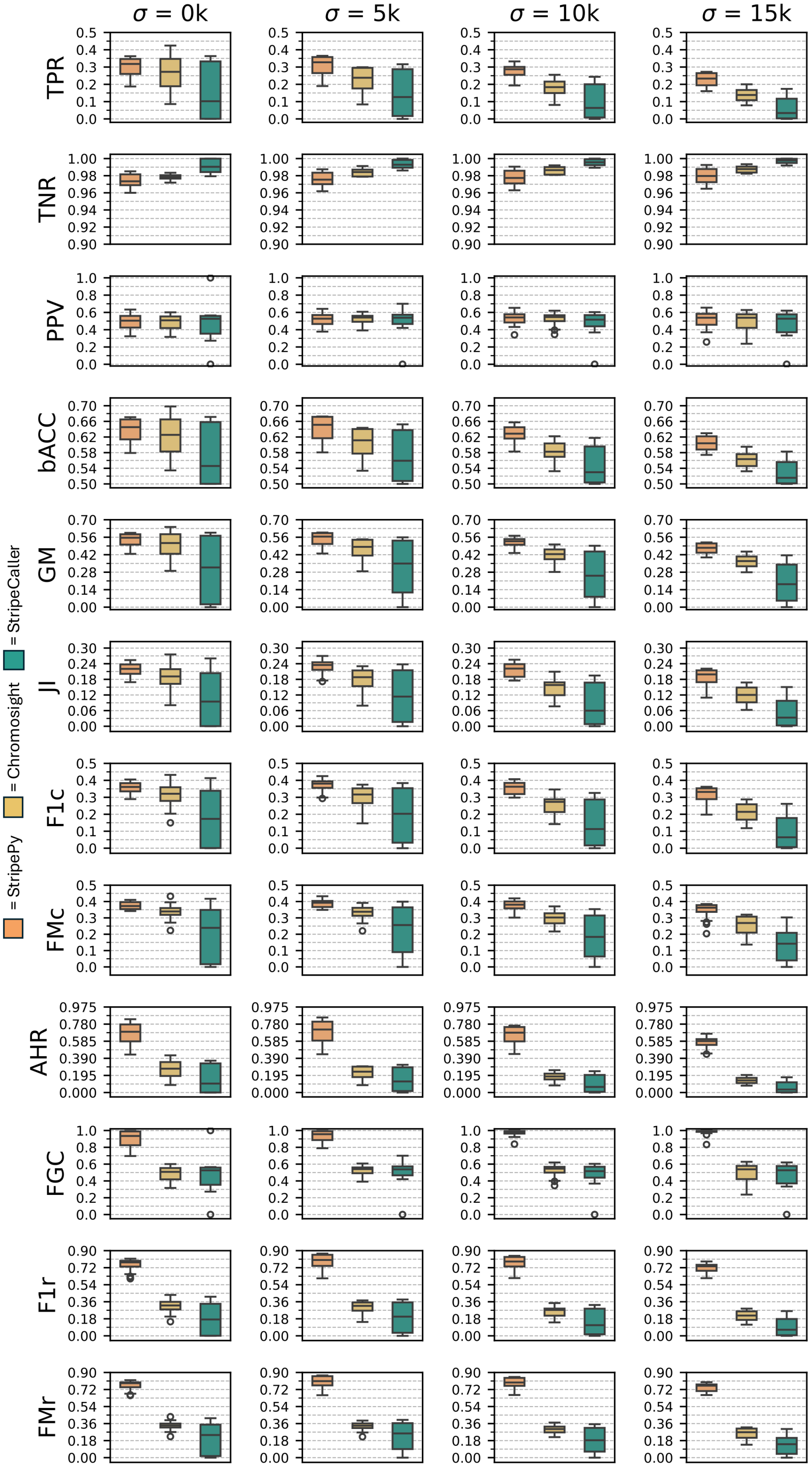

Supplementary Fig. 3. | Change in noise level for the StripeBench's benchmark. Boxplots of the classification (TPR, TNR, PPV, bACC, GM, JI, F1c, and FMc) and recognition (AHR, FGC, F1r, and FMr) measures by change in noise level.

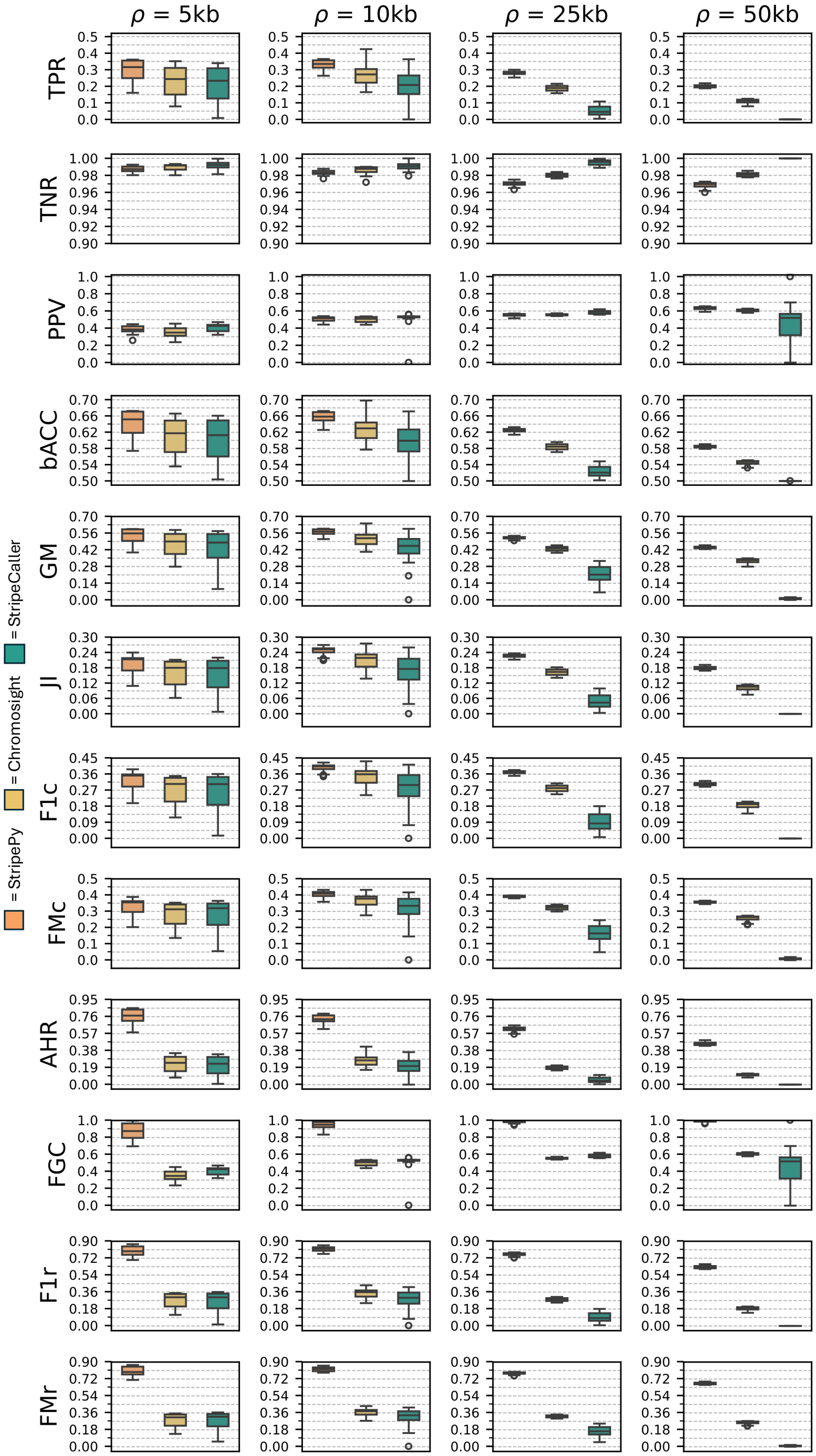

Supplementary Fig. 4. | Change in resolution for the StripeBench's benchmark. Boxplots of the classification (TPR, TNR, PPV, bACC, GM, JI, F1c, and FMc) and recognition (AHR, FGC, F1r, and FMr) measures by change in resolution.

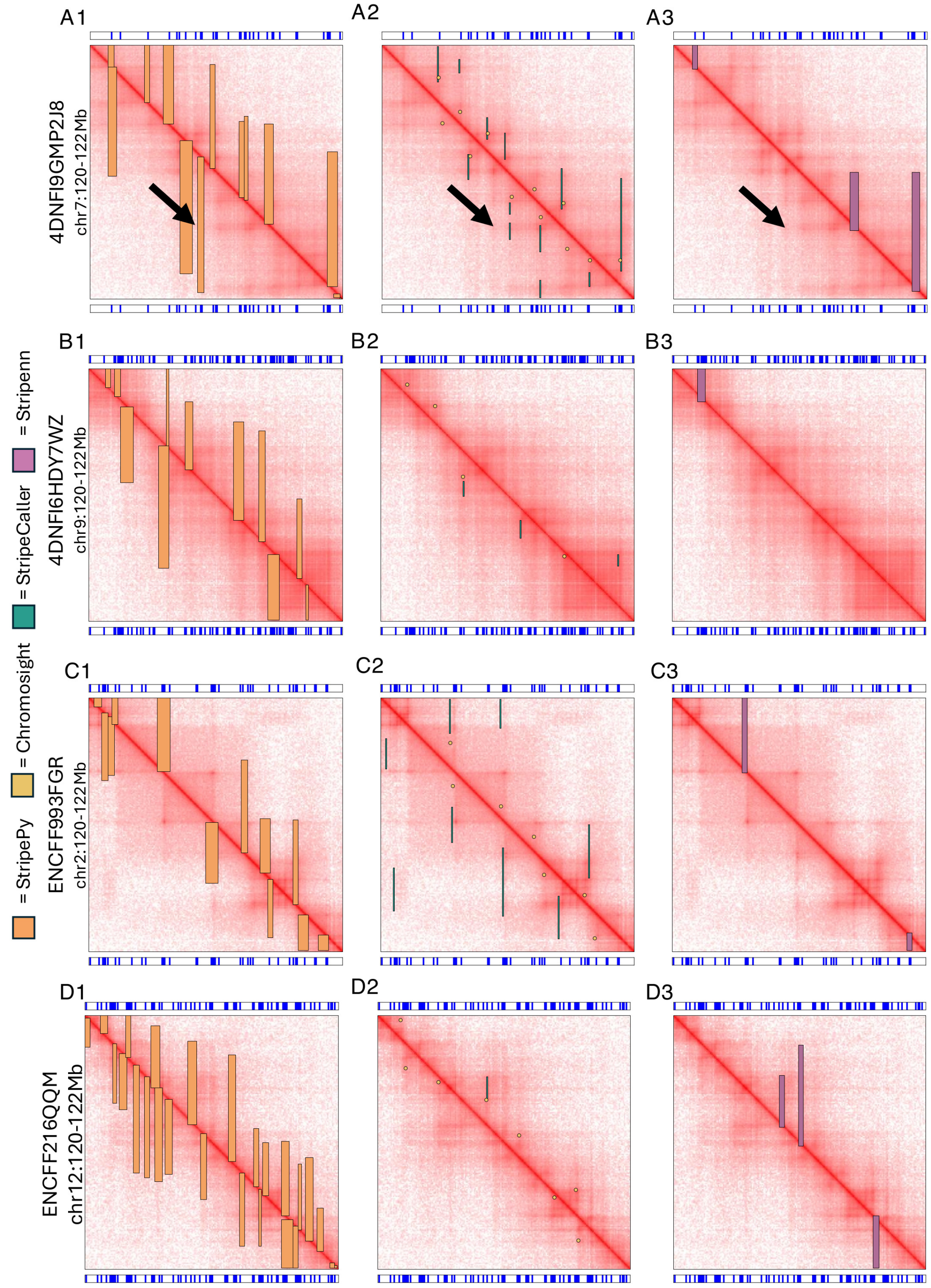

Supplementary Fig. 5. | Four regions from as many contact maps . The upper and lower bands point at bin hosting CTCF sites, obtained from ChIP-Seq data. The images of the contact maps have superimposed the predictions produced by the tools under study. For each map, the region is repeated three times to improve clarity: first column consists of StripePy, second column refers to Chromosight and StripeCaller, last column has Stripenn. In the first row, black arrows highlight an example of mild pattern that is correctly identified by our method but overlooked by the other tools.

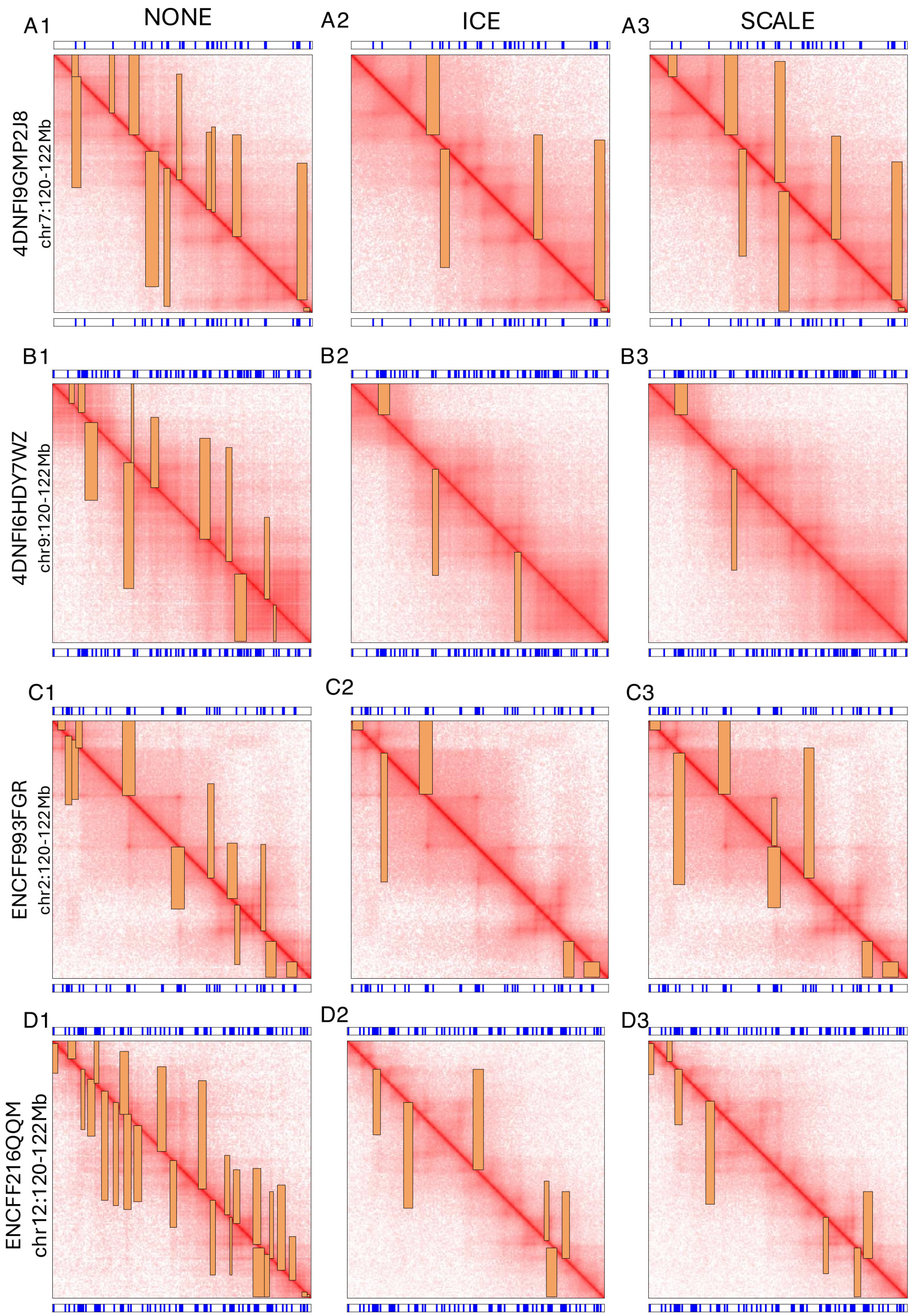

Supplementary Fig. 6. | Effects of normalization. The plots represent four regions from as many contact maps. The upper and lower bands point at bin hosting CTCF sites, obtained from ChIP-Seq data. The images of the contact maps have superimposed the predictions produced by StripePy when applied to three conditions: no normalization (NONE), ICE and SCALE. For each map, the region is repeated three times to improve clarity: from left to right, columns refer to NONE, ICE, and SCALE.

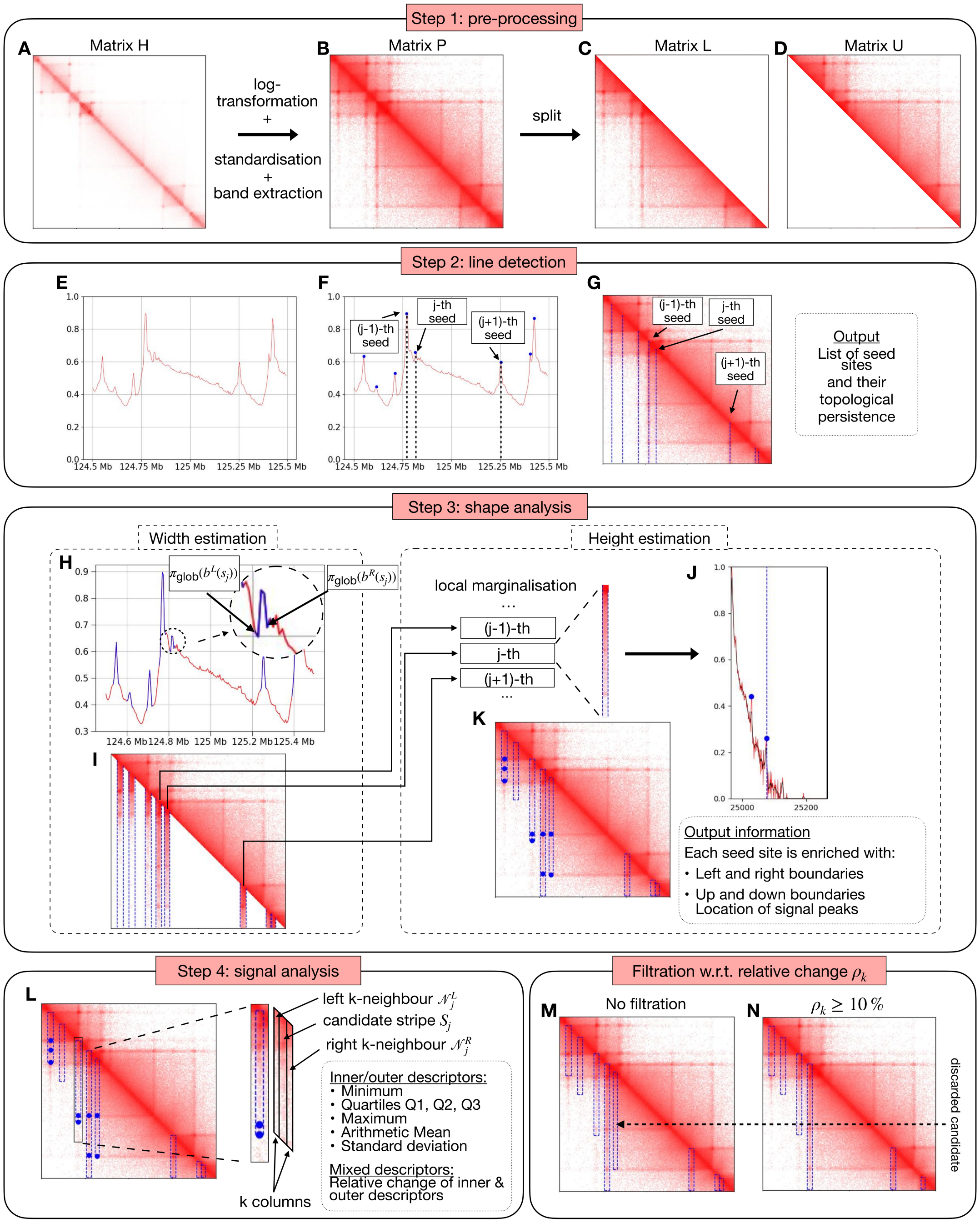

Supplementary Fig. 7. | Extended overview of StripePy. Step 1: **A** region of an input contact map as it is, **B** after global enhancement via log-transformation, and **C-D** final split into lower- and upper-triangular parts; the remaining steps focused on on the lower-triangular part, to keep the plots readable. Step 2: **E** constrained pseudo-distribution obtained by marginalizing L; **F** constrained pseudo-distribution with superimposed persistent maximum points, a.k.a. seeds; **G** pre-processed Hi-C matrix with superimposed vertical lines corresponding to persistent seeds. Step 3: **H** study of neighbourhoods of seed sites allow to introduce notions of left and right boundaries, which can be then used to slice the pre-processed Hi-C matrix **I**; **J** local pseudo-distributions (original: red curve, smoothed: black curve) allow to introduce up/down boundaries (dashed blue line), as well as to detect the location signal peaks (blue dots); **K** overlap rectangles corresponding to candidate stripes, together with location of signal peaks, to the pre-processed Hi-C matrix. Step 4: **L** shows a region neighbouring a candidate stripe and list the sets of inner/outer/mixed descriptors. The initial set of candidate stripes, represented in **M**, is reduced by thresholding one of these descriptors, namely the relative change parameter **N**.

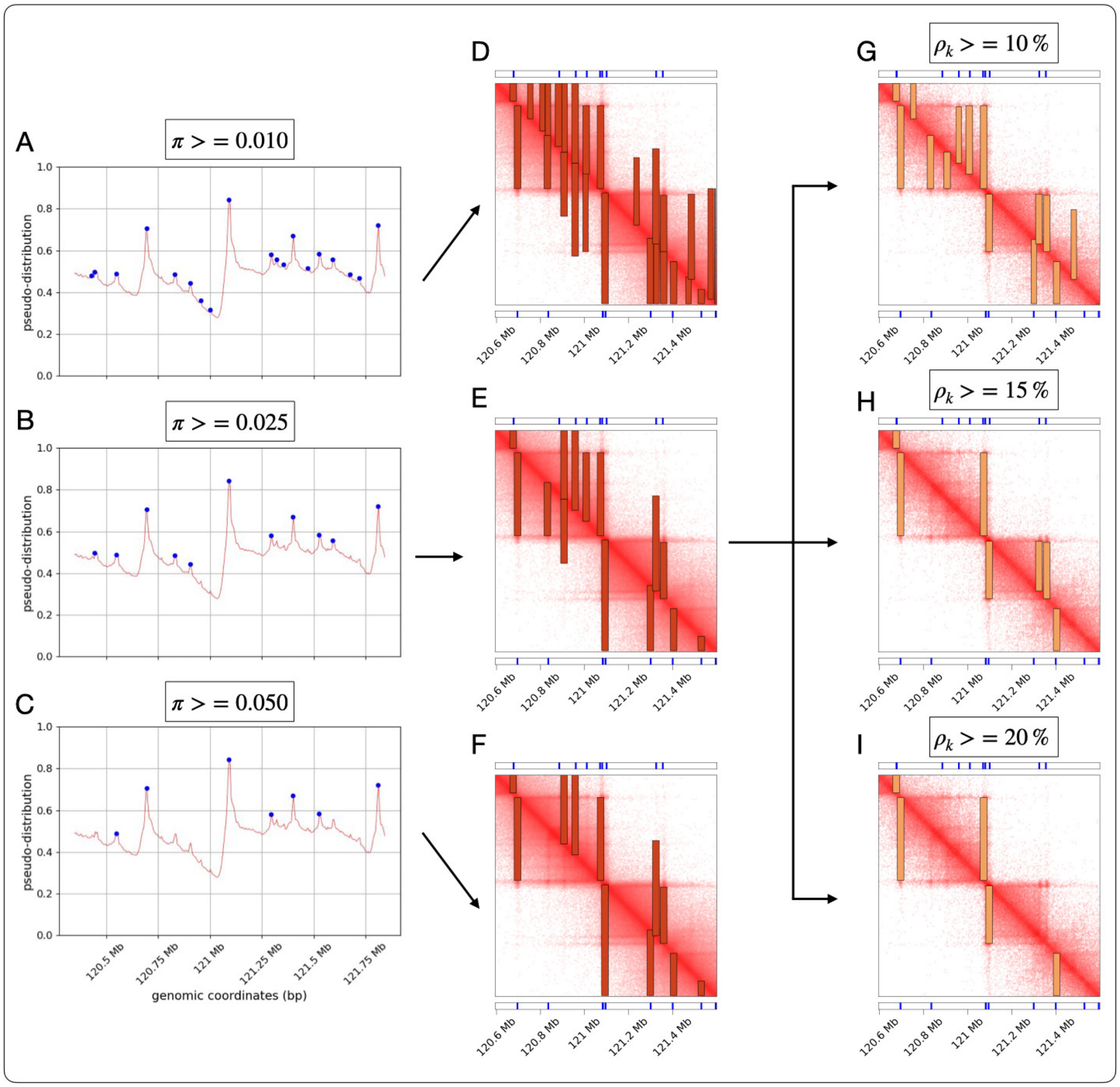

Supplementary Fig. 8. | StripePy's persistence vs. relative change filterings. Topological persistence rules out local maximum points (**A**, **B**, and **C**) in terms of the strength of the linear pattern that each bin exhibit. Three thresholds therefore produce as many collections of candidate stripes (**D**, **E**, and **F**) at the end of Step 3. In Step 4, thresholding relative changes further restricts the number of predicted stripes, this time on the basis of a metric that takes into account a well-defined rectangular region (**G**, **H**, and **I**).

Supplementary Table 1. Number of predicted stripes in the StripeBench benchmark. The following encoding is adopted: M1 = StripePy, M2=Chromosight, M3=StripeCaller, M4=Stripenn.

		**$\sigma = 0kb$**				**$\sigma = 5kb$**				**$\sigma = 10kb$**				**$\sigma = 15kb$**			
**ρ**	**δ**	M1	M2	M3	M4	M1	M2	M3	M4	M1	M2	M3	M4	M1	M2	M3	M4
5kb	1	35,068	30,811	19,877	274	29,868	19,143	12,103	85	24,660	14,666	4,792	37	19,579	10,401	731	1
	5	31,340	33,949	29,251	1,408	26,979	22,190	19,948	139	21,241	15,730	13,577	65	15,732	12,780	6,390	16
	10	29,647	34,619	31,807	2,410	26,478	23,411	22,229	181	20,830	16,029	16,163	84	15,292	12,465	9,501	21
	15	28,629	34,912	33,002	3,092	26,290	23,992	23,612	221	20,565	16,458	17,417	110	15,085	12,414	10,997	22
10kb	1	25,112	21,197	13,662	0	23,277	16,545	9,193	0	20,504	13,204	5,372	0	17,553	10,887	2,357	0
	5	22,266	22,238	19,749	0	21,622	17,428	14,683	0	18,991	14,121	10,965	0	15,919	11,528	7,570	0
	10	21,307	29,460	0	0	21,046	17,760	16,441	0	18,629	14,360	12,638	0	15,567	11,806	9,297	0
	15	20,896	22,708	23,214	1	20,786	17,912	17,359	0	18,437	14,503	13,477	0	15,249	11,959	10,066	0
25kb	1	16,583	11,133	1,691	0	16,181	10,150	1,078	0	15,411	9,070	527	0	14,104	8,036	195	0
	5	15,188	11,291	4,050	0	14,964	10,267	2,967	0	14,460	9,341	1,804	0	13,169	8,250	927	0
	10	14,680	11,327	4,963	0	14,621	10,288	3,804	0	14,127	9,361	2,549	0	12,838	8,335	1,431	0
	15	14,349	11,310	5,518	0	14,433	10,331	4,292	0	13,961	9,373	2,910	0	12,684	8,409	1,732	0
50kb	1	9,519	3,880	1	0	9,370	3,793	1	0	9,331	3,633	0	0	9,031	3,500	0	0
	5	8,387	5,367	11	0	8,298	5,156	8	0	8,337	4,817	10	0	8,212	4,541	3	0
	10	8,018	5,437	23	0	7,984	5,195	13	0	7,982	4,863	11	0	7,875	4,554	7	0
	15	7,763	5,441	26	0	7,758	5,169	20	0	7,758	4,866	19	0	7,705	4,550	13	0

Supplementary Table 2. Classification and recognition measures for each Hi-C matrix in the StripeBench benchmark – resolution set to 5 kb. The following encoding is adopted: M1 = StripePy, M2=Chromosight, M3=StripeCaller, M4=Stripenn.

		$\delta = 1$				$\delta = 5$				$\delta = 10$				$\delta = 15$			
σ	**measure**	M1	M2	M3	M4	M1	M2	M3	M4	M1	M2	M3	M4	M1	M2	M3	M4
0k	TPR	36.13	34.64	28.03	0.51	35.33	35.02	33.53	1.72	34.54	35.12	33.90	2.81	33.95	35.16	33.99	3.51
	TNR	98.03	98.34	99.08	99.99	98.32	98.09	98.45	99.93	98.44	98.04	98.24	99.87	98.51	98.02	98.15	99.83
	PPV	32.37	35.33	44.31	58.03	35.42	32.42	36.02	38.42	36.61	31.88	33.49	36.64	37.26	31.65	32.36	35.64
	bACC	67.08	66.49	63.56	50.25	66.82	66.56	65.99	50.82	66.49	66.58	66.07	51.34	66.23	66.59	66.07	51.67
	GM	59.51	58.37	52.70	7.11	58.94	58.61	57.45	13.12	58.31	58.68	57.71	16.75	57.83	58.71	57.76	18.71
	JI	20.59	21.20	20.73	0.50	21.49	20.24	21.01	1.68	21.62	20.06	20.26	2.68	21.60	19.99	19.87	3.30
	F1c	34.15	34.98	34.34	1.00	35.38	33.67	34.73	3.30	35.55	33.42	33.69	5.22	35.53	33.31	33.15	6.39
	FMc	34.20	34.99	35.24	5.42	35.38	33.69	34.75	8.13	35.56	33.46	33.69	10.15	35.56	33.36	33.16	11.18
	AHR	83.58	34.64	28.03	1.09	81.13	35.02	33.53	5.34	79.45	35.12	33.90	9.06	78.07	35.16	33.99	11.52
	FGC	69.66	35.33	44.31	100.00	75.76	32.42	36.02	100.00	78.39	31.88	33.49	99.96	79.73	31.65	32.36	100.00
	F1r	75.99	34.98	34.34	2.15	78.35	33.67	34.73	10.14	78.92	33.42	33.69	16.62	78.89	33.31	33.15	20.66
	FMr	76.30	34.99	35.24	10.42	78.39	33.69	34.75	23.12	78.92	33.46	33.69	30.10	78.90	33.36	33.16	33.94
5k	TPR	35.91	27.59	18.13	0.14	35.82	29.53	28.78	0.25	35.53	29.78	30.65	0.30	35.57	29.91	31.62	0.43
	TNR	98.46	99.13	99.47	100.00	98.69	98.93	99.09	100.00	98.73	98.83	98.95	99.99	98.74	98.79	98.86	99.99
	PPV	37.78	45.29	47.08	52.94	41.72	41.81	45.33	56.83	42.16	39.98	43.32	52.49	42.52	39.17	42.08	61.54
	bACC	67.18	63.36	58.80	50.07	67.26	64.23	63.93	50.12	67.13	64.31	64.80	50.15	67.16	64.35	65.24	50.21
	GM	59.46	52.30	42.47	3.78	59.46	54.05	53.40	5.01	59.23	54.26	55.07	5.50	59.27	54.36	55.91	6.58
	JI	22.56	20.69	15.06	0.14	23.87	20.93	21.36	0.25	23.89	20.58	21.88	0.30	24.02	20.42	22.03	0.43
	F1c	36.82	34.29	26.18	0.29	38.55	34.61	35.20	0.50	38.56	34.14	35.90	0.60	38.74	33.92	36.10	0.86
	FMc	36.83	35.35	29.22	2.75	38.66	35.14	36.12	3.78	38.70	34.51	36.44	3.98	38.89	34.23	36.47	5.16
	AHR	85.60	27.59	18.13	0.36	85.54	29.53	28.78	0.57	85.30	29.78	30.65	0.76	85.20	29.91	31.62	0.94
	FGC	78.92	45.29	47.08	100.00	86.90	41.81	45.33	100.00	88.24	39.98	43.32	100.00	88.71	39.17	42.08	100.00
	F1r	82.12	34.29	26.18	0.72	86.22	34.61	35.20	1.13	86.74	34.14	35.90	1.51	86.92	33.92	36.10	1.85
	FMr	82.19	35.35	29.22	6.00	86.22	35.14	36.12	7.55	86.76	34.51	36.44	8.72	86.94	34.23	36.47	9.67
10k	TPR	26.72	16.09	6.71	0.05	29.18	19.78	18.94	0.07	29.20	20.45	22.38	0.09	29.22	21.14	24.35	0.11
	TNR	98.65	99.20	99.78	100.00	99.00	99.21	99.37	100.00	99.03	99.20	99.24	100.00	99.05	99.18	99.19	99.99
	PPV	34.05	34.47	43.99	43.24	43.17	39.51	43.83	33.85	44.05	40.09	43.50	32.14	44.65	40.37	43.93	30.00
	bACC	62.69	57.64	53.24	50.02	64.09	59.49	59.15	50.03	64.12	59.83	60.81	50.04	64.14	60.16	61.77	50.05
	GM	51.34	39.95	25.87	2.26	53.75	44.30	43.38	2.65	53.77	45.04	47.12	2.93	53.80	45.79	49.15	3.24
	JI	17.61	12.32	6.18	0.05	21.08	15.18	15.24	0.07	21.30	15.66	17.34	0.09	21.45	16.11	18.58	0.10
	F1c	29.94	21.94	11.64	0.10	34.82	26.36	26.45	0.14	35.12	27.08	29.55	0.17	35.33	27.75	31.33	0.21
	FMc	30.17	23.55	17.18	1.48	35.49	27.95	28.81	1.54	35.86	28.63	31.20	1.66	36.12	29.22	32.71	1.77
	AHR	76.29	16.09	6.71	0.18	75.78	19.78	18.94	0.27	75.18	20.45	22.38	0.37	74.89	21.14	24.35	0.45
	FGC	83.81	34.47	43.99	100.00	95.57	39.51	43.83	100.00	96.38	40.09	43.50	100.00	97.11	40.37	43.93	100.00
	F1r	79.87	21.94	11.64	0.36	84.53	26.36	26.45	0.53	84.47	27.08	29.55	0.74	84.57	27.75	31.33	0.89
	FMr	79.96	23.55	17.18	4.22	85.10	27.95	28.81	5.17	85.12	28.63	31.20	6.10	85.28	29.22	32.71	6.70
15k	TPR	16.06	7.86	0.84	0.00	18.53	10.83	7.45	0.02	19.11	11.47	11.24	0.03	19.51	11.82	13.09	0.03
	TNR	98.79	99.34	99.96	100.00	99.18	99.22	99.66	100.00	99.23	99.26	99.50	100.00	99.26	99.28	99.43	100.00
	PPV	25.77	23.74	36.25	100.00	37.02	26.64	36.64	37.50	39.28	28.91	37.19	42.86	40.64	29.93	37.39	45.45
	bACC	57.43	53.60	50.40	50.00	58.86	55.03	53.56	50.01	59.17	55.37	55.37	50.01	59.38	55.55	56.26	50.02
	GM	39.83	27.94	9.18	0.56	42.87	32.78	27.25	1.38	43.55	33.74	33.45	1.69	44.00	34.26	36.07	1.78
	JI	10.98	6.27	0.83	0.00	14.09	8.34	6.60	0.02	14.75	8.95	9.45	0.03	15.18	9.26	10.73	0.03
	F1c	19.79	11.81	1.65	0.01	24.70	15.40	12.38	0.04	25.71	16.42	17.27	0.06	26.36	16.95	19.39	0.06
	FMc	20.34	13.66	5.53	0.56	26.19	16.99	16.52	0.85	27.40	18.21	20.45	1.11	28.16	18.81	22.12	1.20
	AHR	60.51	7.86	0.84	0.00	59.80	10.83	7.45	0.07	58.87	11.47	11.24	0.11	58.44	11.82	13.09	0.12
	FGC	83.29	23.74	36.25	100.00	98.94	26.64	36.64	100.00	99.61	28.91	37.19	100.00	99.72	29.93	37.39	100.00
	F1r	70.09	11.81	1.65	0.01	74.54	15.40	12.38	0.15	74.01	16.42	17.27	0.22	73.70	16.95	19.39	0.25
	FMr	70.99	13.66	5.53	0.56	76.92	16.99	16.52	2.71	76.58	18.21	20.45	3.34	76.34	18.81	22.12	3.52

Supplementary Table 3. Classification and recognition measures for each Hi-C matrix in the StripeBench benchmark – resolution set to 10 kb. The following encoding is adopted: M1 = StripePy, M2=Chromosight, M3=StripeCaller, M4=Stripenn.

		$\delta = 1$				$\delta = 5$				$\delta = 10$				$\delta = 15$			
σ	**measure**	M1	M2	M3	M4	M1	M2	M3	M4	M1	M2	M3	M4	M1	M2	M3	M4
0k	TPR	36.27	32.93	24.10	0.00	35.01	33.54	33.17	0.00	34.10	42.43	0.00	0.00	33.69	33.75	36.33	0.00
	TNR	97.61	98.10	98.93	100.00	98.03	97.96	98.36	100.00	98.15	97.19	100.00	100.00	98.20	97.89	97.94	100.00
	PPV	44.17	47.51	53.95	0.00	48.08	46.13	51.36	0.00	48.94	44.04	0.00	0.00	49.30	45.45	47.86	100.00
	bACC	66.94	65.52	61.52	50.00	66.52	65.75	65.77	50.00	66.12	69.81	50.00	50.00	65.94	65.82	67.14	50.00
	GM	59.50	56.84	48.83	0.00	58.58	57.32	57.12	0.00	57.85	64.22	0.00	0.00	57.51	57.48	59.65	0.57
	JI	24.87	24.14	19.99	0.00	25.40	24.10	25.24	0.00	25.15	27.57	0.00	0.00	25.02	24.02	26.03	0.00
	F1c	39.84	38.90	33.32	0.00	40.51	38.84	40.31	0.00	40.19	43.22	0.00	0.00	40.03	38.73	41.31	0.01
	FMc	40.03	39.55	36.06	0.00	41.02	39.34	41.27	0.00	40.85	43.23	0.00	0.00	40.75	39.16	41.70	0.57
	AHR	77.56	32.93	24.10	0.00	74.57	33.54	33.17	0.00	72.98	42.43	0.00	0.00	72.33	33.75	36.33	0.01
	FGC	83.39	47.51	53.95	0.00	89.91	46.13	51.36	0.00	91.73	44.04	0.00	0.00	92.73	45.45	47.86	100.00
	F1r	80.37	38.90	33.32	0.00	81.53	38.84	40.31	0.00	81.28	43.22	0.00	0.00	81.27	38.73	41.31	0.01
	FMr	80.42	39.55	36.06	0.00	81.88	39.34	41.27	0.00	81.82	43.23	0.00	0.00	81.90	39.16	41.70	0.81
5k	TPR	36.49	28.81	16.75	0.00	36.27	29.39	25.81	0.00	35.76	29.57	28.74	0.00	35.67	29.57	30.09	0.00
	TNR	97.94	98.68	99.31	100.00	98.21	98.56	98.84	100.00	98.28	98.52	98.70	100.00	98.32	98.49	98.61	100.00
	PPV	47.94	53.25	55.71	0.00	51.30	51.58	53.76	0.00	51.96	50.92	53.46	0.00	52.47	50.49	53.01	0.00
	bACC	67.21	63.75	58.03	50.00	67.24	63.98	62.33	50.00	67.02	64.04	63.72	50.00	66.99	64.03	64.35	50.00
	GM	59.78	53.32	40.78	0.00	59.69	53.83	50.51	0.00	59.28	53.97	53.26	0.00	59.22	53.97	54.47	0.00
	JI	26.13	23.00	14.78	0.00	26.98	23.04	21.12	0.00	26.87	23.01	22.99	0.00	26.96	22.92	23.75	0.00
	F1c	41.44	37.39	25.75	0.00	42.50	37.45	34.88	0.00	42.36	37.41	37.39	0.00	42.47	37.30	38.39	0.00
	FMc	41.82	39.17	30.54	0.00	43.14	38.94	37.25	0.00	43.10	38.80	39.20	0.00	43.26	38.64	39.94	0.00
	AHR	79.38	28.81	16.75	0.00	78.44	29.39	25.81	0.00	77.74	29.57	28.74	0.00	77.42	29.57	30.09	0.00
	FGC	88.47	53.25	55.71	0.00	93.65	51.58	53.76	0.00	95.29	50.92	53.46	0.00	95.97	50.49	53.01	0.00
	F1r	83.67	37.39	25.75	0.00	85.38	37.45	34.88	0.00	85.63	37.41	37.39	0.00	85.71	37.30	38.39	0.00
	FMr	83.80	39.17	30.54	0.00	85.71	38.94	37.25	0.00	86.07	38.80	39.20	0.00	86.20	38.64	39.94	0.00
10k	TPR	33.26	22.91	9.85	0.00	32.86	24.81	19.32	0.00	32.74	25.25	22.25	0.00	32.57	25.52	23.48	0.00
	TNR	98.24	98.94	99.60	100.00	98.48	98.89	99.14	100.00	98.53	98.87	99.01	100.00	98.56	98.86	98.93	100.00
	PPV	49.61	53.07	56.07	0.00	52.91	53.73	53.88	0.00	53.74	53.78	53.83	0.00	54.02	53.81	53.27	0.00
	bACC	65.75	60.93	54.72	50.00	65.67	61.85	59.23	50.00	65.64	62.06	60.63	50.00	65.56	62.19	61.20	50.00
	GM	57.16	47.61	31.32	0.00	56.89	49.53	43.76	0.00	56.80	49.97	46.93	0.00	56.66	50.23	48.19	0.00
	JI	24.86	19.05	9.14	0.00	25.43	20.44	16.58	0.00	25.54	20.75	18.68	0.00	25.50	20.93	19.47	0.00
	F1c	39.82	32.01	16.76	0.00	40.54	33.94	28.44	0.00	40.69	34.37	31.48	0.00	40.64	34.62	32.59	0.00
	FMc	40.62	34.87	23.50	0.00	41.70	36.51	32.26	0.00	41.95	36.85	34.60	0.00	41.95	37.06	35.36	0.00
	AHR	74.63	22.91	9.85	0.00	73.19	24.81	19.32	0.00	72.45	25.25	22.25	0.00	72.05	25.52	23.48	0.00
	FGC	92.29	53.07	56.07	0.00	97.30	53.73	53.88	0.00	98.04	53.78	53.83	0.00	98.47	53.81	53.27	0.00
	F1r	82.52	32.01	16.76	0.00	83.54	33.94	28.44	0.00	83.33	34.37	31.48	0.00	83.21	34.62	32.59	0.00
	FMr	82.99	34.87	23.50	0.00	84.39	36.51	32.26	0.00	84.28	36.85	34.60	0.00	84.23	37.06	35.36	0.00
15k	TPR	27.19	16.46	4.04	0.00	27.24	18.62	13.03	0.00	26.79	19.65	16.04	0.00	26.37	20.01	17.37	0.00
	TNR	98.43	99.00	99.81	100.00	98.71	99.01	99.39	100.00	98.74	99.01	99.25	100.00	98.78	99.01	99.19	100.00
	PPV	47.37	46.24	52.48	0.00	52.32	49.39	52.64	0.00	52.63	50.90	52.77	0.00	52.88	51.16	52.78	0.00
	bACC	62.81	57.73	51.93	50.00	62.97	58.81	56.21	50.00	62.77	59.33	57.65	50.00	62.57	59.51	58.28	50.00
	GM	51.73	40.37	20.09	0.00	51.85	42.94	35.99	0.00	51.43	44.11	39.90	0.00	51.03	44.50	41.51	0.00
	JI	20.88	13.82	3.90	0.00	21.82	15.64	11.66	0.00	21.59	16.52	14.03	0.00	21.35	16.80	15.04	0.00
	F1c	34.55	24.28	7.51	0.00	35.82	27.04	20.89	0.00	35.51	28.35	24.61	0.00	35.19	28.76	26.14	0.00
	FMc	35.89	27.59	14.57	0.00	37.75	30.33	26.19	0.00	37.55	31.62	29.10	0.00	37.34	31.99	30.28	0.00
	AHR	67.04	16.46	4.04	0.00	63.98	18.62	13.03	0.00	63.17	19.65	16.04	0.00	62.19	20.01	17.37	0.00
	FGC	94.91	46.24	52.48	0.00	99.05	49.39	52.64	0.00	99.43	50.90	52.77	0.00	99.61	51.16	52.78	0.00
	F1r	78.57	24.28	7.51	0.00	77.74	27.04	20.89	0.00	77.25	28.35	24.61	0.00	76.57	28.76	26.14	0.00
	FMr	79.76	27.59	14.57	0.00	79.60	30.33	26.19	0.00	79.25	31.62	29.10	0.00	78.71	31.99	30.28	0.00

Supplementary Table 4. Classification and recognition measures for each Hi-C matrix in the StripeBench benchmark – resolution set to 25 kb. The following encoding is adopted: M1 = StripePy, M2=Chromosight, M3=StripeCaller, M4=Stripenn.

		$\delta = 1$				$\delta = 5$				$\delta = 10$				$\delta = 15$			
σ	**measure**	M1	M2	M3	M4	M1	M2	M3	M4	M1	M2	M3	M4	M1	M2	M3	M4
0k	TPR	29.89	21.04	3.30	0.00	28.81	21.49	7.98	0.00	27.99	21.54	9.66	0.00	27.52	21.46	10.80	0.00
	TNR	96.33	97.66	99.66	100.00	96.82	97.65	99.19	100.00	96.95	97.64	98.99	100.00	97.04	97.64	98.89	100.00
	PPV	51.61	54.10	55.82	0.00	54.31	54.50	56.44	0.00	54.60	54.44	55.73	0.00	54.90	54.33	56.03	0.00
	bACC	63.11	59.35	51.48	50.00	62.82	59.57	53.59	50.00	62.47	59.59	54.33	50.00	62.28	59.55	54.84	50.00
	GM	53.66	45.33	18.13	0.00	52.81	45.81	28.14	0.00	52.10	45.86	30.93	0.00	51.67	45.78	32.68	0.00
	JI	23.35	17.85	3.21	0.00	23.19	18.22	7.52	0.00	22.71	18.25	8.97	0.00	22.44	18.18	9.96	0.00
	F1c	37.86	30.29	6.23	0.00	37.65	30.83	13.99	0.00	37.01	30.86	16.47	0.00	36.66	30.77	18.11	0.00
	FMc	39.28	33.74	13.57	0.00	39.55	34.23	21.23	0.00	39.10	34.24	23.20	0.00	38.87	34.15	24.60	0.00
	AHR	66.28	21.04	3.30	0.00	63.85	21.49	7.98	0.00	62.32	21.54	9.66	0.00	61.36	21.46	10.80	0.00
	FGC	94.28	54.10	55.82	0.00	98.29	54.50	56.44	0.00	98.86	54.44	55.73	0.00	99.16	54.33	56.03	0.00
	F1r	77.84	30.29	6.23	0.00	77.41	30.83	13.99	0.00	76.44	30.86	16.47	0.00	75.81	30.77	18.11	0.00
	FMr	79.05	33.74	13.57	0.00	79.22	34.23	21.23	0.00	78.49	34.24	23.20	0.00	78.00	34.15	24.60	0.00
5k	TPR	29.99	19.49	2.22	0.00	28.80	19.89	5.96	0.00	28.46	19.91	7.61	0.00	28.15	20.01	8.52	0.00
	TNR	96.52	97.91	99.80	100.00	96.92	97.91	99.42	100.00	97.04	97.90	99.26	100.00	97.08	97.89	99.15	100.00
	PPV	53.07	54.97	59.00	0.00	55.10	55.47	57.47	0.00	55.72	55.40	57.26	0.00	55.84	55.45	56.85	0.00
	bACC	63.26	58.70	51.01	50.00	62.86	58.90	52.69	50.00	62.75	58.90	53.43	50.00	62.61	58.95	53.84	50.00
	GM	53.80	43.68	14.89	0.00	52.83	44.13	24.33	0.00	52.55	44.15	27.48	0.00	52.27	44.26	29.07	0.00
	JI	23.70	16.80	2.19	0.00	23.32	17.15	5.70	0.00	23.21	17.16	7.20	0.00	23.02	17.24	8.00	0.00
	F1c	38.32	28.77	4.28	0.00	37.83	29.28	10.79	0.00	37.67	29.29	13.43	0.00	37.43	29.41	14.82	0.00
	FMc	39.90	32.73	11.45	0.00	39.83	33.22	18.50	0.00	39.82	33.21	20.87	0.00	39.64	33.31	22.01	0.00
	AHR	66.29	19.49	2.22	0.00	64.07	19.89	5.96	0.00	63.08	19.91	7.61	0.00	62.53	20.01	8.52	0.00
	FGC	95.33	54.97	59.00	0.00	98.80	55.47	57.47	0.00	99.26	55.40	57.26	0.00	99.43	55.45	56.85	0.00
	F1r	78.20	28.77	4.28	0.00	77.73	29.28	10.79	0.00	77.14	29.29	13.43	0.00	76.78	29.41	14.82	0.00
	FMr	79.50	32.73	11.45	0.00	79.56	33.22	18.50	0.00	79.13	33.21	20.87	0.00	78.85	33.31	22.01	0.00
10k	TPR	29.27	17.58	1.11	0.00	28.24	18.29	3.77	0.00	27.89	18.38	5.34	0.00	27.64	18.34	6.02	0.00
	TNR	96.78	98.15	99.90	100.00	97.08	98.12	99.67	100.00	97.19	98.12	99.53	100.00	97.23	98.11	99.46	100.00
	PPV	54.37	55.49	60.34	0.00	55.91	56.08	59.87	0.00	56.53	56.22	59.95	0.00	56.69	56.01	59.21	0.00
	bACC	63.02	57.87	50.51	50.00	62.66	58.21	51.72	50.00	62.54	58.25	52.43	50.00	62.44	58.22	52.74	50.00
	GM	53.22	41.54	10.53	0.00	52.36	42.37	19.39	0.00	52.07	42.47	23.05	0.00	51.85	42.42	24.46	0.00
	JI	23.49	15.41	1.10	0.00	23.09	16.00	3.68	0.00	22.97	16.08	5.15	0.00	22.82	16.03	5.78	0.00
	F1c	38.05	26.70	2.18	0.00	37.52	27.59	7.10	0.00	37.35	27.71	9.80	0.00	37.17	27.63	10.93	0.00
	FMc	39.89	31.23	8.19	0.00	39.73	32.03	15.03	0.00	39.71	32.15	17.89	0.00	39.59	32.05	18.88	0.00
	AHR	64.49	17.58	1.11	0.00	62.75	18.29	3.77	0.00	61.63	18.38	5.34	0.00	61.21	18.34	6.02	0.00
	FGC	96.20	55.49	60.34	0.00	99.09	56.08	59.87	0.00	99.47	56.22	59.95	0.00	99.65	56.01	59.21	0.00
	F1r	77.22	26.70	2.18	0.00	76.84	27.59	7.10	0.00	76.10	27.71	9.80	0.00	75.84	27.63	10.93	0.00
	FMr	78.77	31.23	8.19	0.00	78.85	32.03	15.03	0.00	78.29	32.15	17.89	0.00	78.10	32.05	18.88	0.00
15k	TPR	27.02	15.86	0.41	0.00	26.00	16.49	2.00	0.00	25.54	16.70	3.10	0.00	25.30	16.80	3.68	0.00
	TNR	97.08	98.40	99.96	100.00	97.38	98.38	99.84	100.00	97.47	98.37	99.75	100.00	97.51	98.35	99.69	100.00
	PPV	54.85	56.51	59.49	0.00	56.53	57.21	61.70	0.00	56.96	57.35	62.05	0.00	57.10	57.21	60.91	0.00
	bACC	62.05	57.13	50.18	50.00	61.69	57.43	50.92	50.00	61.50	57.53	51.43	50.00	61.40	57.58	51.69	50.00
	GM	51.22	39.51	6.36	0.00	50.32	40.27	14.12	0.00	49.89	40.53	17.59	0.00	49.67	40.65	19.17	0.00
	JI	22.10	14.13	0.40	0.00	21.67	14.68	1.97	0.00	21.41	14.85	3.04	0.00	21.26	14.93	3.60	0.00
	F1c	36.20	24.77	0.80	0.00	35.62	25.60	3.87	0.00	35.26	25.86	5.91	0.00	35.06	25.98	6.95	0.00
	FMc	38.50	29.94	4.91	0.00	38.34	30.71	11.10	0.00	38.14	30.94	13.87	0.00	38.01	31.01	14.98	0.00
	AHR	59.94	15.86	0.41	0.00	58.01	16.49	2.00	0.00	56.78	16.70	3.10	0.00	56.22	16.80	3.68	0.00
	FGC	96.87	56.51	59.49	0.00	99.38	57.21	61.70	0.00	99.64	57.35	62.05	0.00	99.79	57.21	60.91	0.00
	F1r	74.06	24.77	0.80	0.00	73.26	25.60	3.87	0.00	72.34	25.86	5.91	0.00	71.92	25.98	6.95	0.00
	FMr	76.20	29.94	4.91	0.00	75.93	30.71	11.10	0.00	75.22	30.94	13.87	0.00	74.90	31.01	14.98	0.00

Supplementary Table 5. Classification and recognition measures for each Hi-C matrix in the StripeBench benchmark – resolution set to 50 kb. The following encoding is adopted: M1 = StripePy, M2=Chromosight, M3=StripeCaller, M4=Stripenn.

		$\delta = 1$				$\delta = 5$				$\delta = 10$				$\delta = 15$			
σ	**measure**	M1	M2	M3	M4	M1	M2	M3	M4	M1	M2	M3	M4	M1	M2	M3	M4
0k	TPR	21.48	8.59	0.00	0.00	19.97	12.26	0.01	0.00	19.16	12.47	0.05	0.00	18.76	12.47	0.05	0.00
	TNR	96.00	98.32	100.00	100.00	96.76	97.79	99.99	100.00	96.92	97.77	99.99	100.00	97.07	97.77	99.99	100.00
	PPV	59.09	57.96	100.00	0.00	62.35	59.83	27.27	0.00	62.58	60.09	56.52	0.00	63.30	60.04	53.85	0.00
	bACC	58.74	53.46	50.00	50.00	58.36	55.02	50.00	50.00	58.04	55.12	50.02	50.00	57.92	55.12	50.02	50.00
	GM	45.41	29.06	0.62	0.00	43.95	34.62	1.07	0.00	43.09	34.92	2.23	0.00	42.68	34.92	2.31	0.00
	JI	18.70	8.08	0.00	0.00	17.82	11.33	0.01	0.00	17.19	11.52	0.05	0.00	16.92	11.52	0.05	0.00
	F1c	31.50	14.96	0.01	0.00	30.25	20.35	0.02	0.00	29.34	20.66	0.10	0.00	28.95	20.66	0.11	0.00
	FMc	35.63	22.31	0.62	0.00	35.28	27.08	0.56	0.00	34.63	27.38	1.67	0.00	34.46	27.37	1.70	0.00
	AHR	49.38	8.59	0.00	0.00	46.00	12.26	0.01	0.00	44.32	12.47	0.05	0.00	43.32	12.47	0.05	0.00
	FGC	95.85	57.96	100.00	0.00	99.24	59.83	27.27	0.00	99.35	60.09	56.52	0.00	99.65	60.04	53.85	0.00
	F1r	65.18	14.96	0.01	0.00	62.87	20.35	0.02	0.00	61.29	20.66	0.10	0.00	60.38	20.66	0.11	0.00
	FMr	68.80	22.31	0.62	0.00	67.57	27.08	0.56	0.00	66.35	27.38	1.67	0.00	65.70	27.37	1.70	0.00
5k	TPR	21.60	8.39	0.00	0.00	19.99	11.82	0.02	0.00	19.48	11.95	0.03	0.00	19.00	11.99	0.05	0.00
	TNR	96.19	98.36	100.00	100.00	96.85	97.88	100.00	100.00	97.04	97.88	99.99	100.00	97.14	97.92	99.99	100.00
	PPV	60.37	57.92	0.00	0.00	63.10	60.03	62.50	0.00	63.90	60.25	53.85	0.00	64.13	60.75	70.00	0.00
	bACC	58.89	53.37	50.00	50.00	58.42	54.85	50.01	50.00	58.26	54.92	50.01	50.00	58.07	54.95	50.02	50.00
	GM	45.58	28.72	0.00	0.00	44.00	34.01	1.38	0.00	43.48	34.20	1.63	0.00	42.96	34.26	2.31	0.00
	JI	18.92	7.91	0.00	0.00	17.90	10.96	0.02	0.00	17.55	11.08	0.03	0.00	17.17	11.13	0.05	0.00
	F1c	31.82	14.65	0.00	0.00	30.36	19.75	0.04	0.00	29.86	19.95	0.05	0.00	29.31	20.03	0.11	0.00
	FMc	36.11	22.04	0.00	0.00	35.52	26.63	1.09	0.00	35.28	26.83	1.20	0.00	34.90	26.99	1.93	0.00
	AHR	49.31	8.39	0.00	0.00	45.98	11.82	0.02	0.00	44.66	11.95	0.03	0.00	43.68	11.99	0.05	0.00
	FGC	96.45	57.92	0.00	0.00	99.29	60.03	62.50	0.00	99.62	60.25	53.85	0.00	99.73	60.75	70.00	0.00
	F1r	65.26	14.65	0.00	0.00	62.85	19.75	0.04	0.00	61.68	19.95	0.05	0.00	60.75	20.03	0.11	0.00
	FMr	68.97	22.04	0.00	0.00	67.57	26.63	1.09	0.00	66.71	26.83	1.20	0.00	66.00	26.99	1.93	0.00
10k	TPR	21.85	8.08	0.00	0.00	20.41	11.33	0.02	0.00	19.72	11.45	0.02	0.00	19.34	11.52	0.03	0.00
	TNR	96.29	98.44	100.00	100.00	96.93	98.10	99.99	100.00	97.11	98.08	99.99	100.00	97.23	98.10	99.99	100.00
	PPV	61.33	58.24	0.00	0.00	64.12	61.62	50.00	0.00	64.71	61.65	45.45	0.00	65.27	61.98	36.84	0.00
	bACC	59.07	53.26	50.00	50.00	58.67	54.72	50.01	50.00	58.41	54.77	50.01	50.00	58.28	54.81	50.01	50.00
	GM	45.87	28.20	0.00	0.00	44.48	33.34	1.38	0.00	43.76	33.51	1.38	0.00	43.36	33.61	1.63	0.00
	JI	19.21	7.64	0.00	0.00	18.32	10.59	0.02	0.00	17.81	10.69	0.02	0.00	17.53	10.76	0.03	0.00
	F1c	32.22	14.19	0.00	0.00	30.97	19.14	0.04	0.00	30.23	19.31	0.04	0.00	29.83	19.42	0.05	0.00
	FMc	36.61	21.69	0.00	0.00	36.18	26.42	0.98	0.00	35.72	26.57	0.93	0.00	35.53	26.72	0.99	0.00
	AHR	49.70	8.08	0.00	0.00	46.47	11.33	0.02	0.00	45.03	11.45	0.02	0.00	43.93	11.52	0.03	0.00
	FGC	96.67	58.24	0.00	0.00	99.47	61.62	50.00	0.00	99.69	61.65	45.45	0.00	99.81	61.98	36.84	0.00
	F1r	65.65	14.19	0.00	0.00	63.35	19.14	0.04	0.00	62.04	19.31	0.04	0.00	61.01	19.42	0.05	0.00
	FMr	69.32	21.69	0.00	0.00	67.99	26.42	0.98	0.00	67.00	26.56	0.93	0.00	66.22	26.72	0.99	0.00
15k	TPR	21.39	7.93	0.00	0.00	20.23	10.79	0.00	0.00	19.57	10.84	0.02	0.00	19.26	10.91	0.03	0.00
	TNR	96.48	98.54	100.00	100.00	97.01	98.24	100.00	100.00	97.18	98.24	100.00	100.00	97.27	98.26	99.99	100.00
	PPV	62.02	59.37	0.00	0.00	64.50	62.23	33.33	0.00	65.09	62.32	57.14	0.00	65.48	62.79	53.85	0.00
	bACC	58.93	53.24	50.00	50.00	58.62	54.51	50.00	50.00	58.37	54.54	50.01	50.00	58.27	54.58	50.01	50.00
	GM	45.42	27.96	0.00	0.00	44.29	32.56	0.62	0.00	43.61	32.63	1.24	0.00	43.29	32.74	1.63	0.00
	JI	18.91	7.53	0.00	0.00	18.20	10.13	0.00	0.00	17.71	10.17	0.02	0.00	17.49	10.25	0.03	0.00
	F1c	31.80	14.00	0.00	0.00	30.79	18.39	0.01	0.00	30.10	18.46	0.03	0.00	29.77	18.59	0.05	0.00
	FMc	36.42	21.70	0.00	0.00	36.12	25.91	0.36	0.00	35.69	25.99	0.93	0.00	35.51	26.17	1.20	0.00
	AHR	48.58	7.93	0.00	0.00	46.02	10.79	0.00	0.00	44.53	10.84	0.02	0.00	43.83	10.91	0.03	0.00
	FGC	96.78	59.37	0.00	0.00	99.55	62.23	33.33	0.00	99.77	62.32	57.14	0.00	99.86	62.79	53.85	0.00
	F1r	64.69	14.00	0.00	0.00	62.95	18.39	0.01	0.00	61.58	18.46	0.03	0.00	60.92	18.59	0.05	0.00
	FMr	68.57	21.70	0.00	0.00	67.69	25.91	0.36	0.00	66.65	25.99	0.93	0.00	66.16	26.17	1.20	0.00

Supplementary Table 6. Clamped p-values for the Anderson-Darling test run over sub-sets of the StripeBench benchmark (obtained by fixing one between contact density δ, noise level σ, and resolution ρ) and the whole benchmark. The minimum value used for clamping is set to $1e-04$. Values below the significant threshold of 0.05 are written in bold.

		methods	TPR	TNR	PPV	bACC	GM	F1C	FMc	JI	AHR	FGC	F1r	FMr
contact density δ	1	M1, M2	**2.2e-03**	**1.3e-03**	6.0e-01	**6.5e-03**	**2.4e-03**	**3.4e-03**	**3.4e-03**	**5.0e-04**	**1.0e-04**	**1.0e-04**	**1.0e-04**	**1.0e-04**
		M1, M3	**1.0e-04**	**1.0e-04**	6.3e-01	**1.0e-04**	**1.0e-04**	**1.0e-04**	**1.0e-04**	**1.0e-04**	**1.0e-04**	**1.0e-04**	**1.0e-04**	**1.0e-04**
		M2, M3	**1.1e-03**	**1.0e-04**	4.2e-01	**1.4e-03**	**1.1e-03**	**1.0e-03**	**1.0e-03**	**1.6e-03**	**1.1e-03**	4.2e-01	**1.0e-03**	**1.6e-03**
	5	M1, M2	**7.0e-03**	5.0e-02	6.4e-01	**3.0e-02**	**1.5e-02**	**1.2e-03**	**1.2e-03**	**3.0e-04**	**1.0e-04**	**1.0e-04**	**1.0e-04**	**1.0e-04**
		M1, M3	**5.0e-04**	**1.0e-04**	5.1e-01	**1.5e-03**	**7.0e-04**	**3.0e-04**	**3.0e-04**	**2.0e-04**	**1.0e-04**	**1.0e-04**	**1.0e-04**	**1.0e-04**
		M2, M3	**1.8e-02**	**1.0e-04**	9.6e-01	**2.3e-02**	**2.0e-02**	**2.1e-02**	**2.1e-02**	**2.1e-02**	**1.8e-02**	9.6e-01	**2.1e-02**	**2.1e-02**
	10	M1, M2	**4.0e-02**	8.2e-02	5.8e-01	5.2e-02	**4.7e-02**	**2.9e-03**	**2.9e-03**	**4.0e-04**	**1.0e-04**	**1.0e-04**	**1.0e-04**	**1.0e-04**
		M1, M3	**9.0e-04**	**1.0e-04**	5.5e-01	**9.0e-04**	**9.0e-04**	**3.0e-04**	**3.0e-04**	**1.0e-04**	**1.0e-04**	**1.0e-04**	**1.0e-04**	**1.0e-04**
		M2, M3	**8.5e-03**	**2.0e-04**	9.2e-01	**2.0e-02**	**8.5e-03**	**1.0e-02**	**1.0e-02**	**1.4e-02**	**8.5e-03**	9.2e-01	**1.0e-02**	**1.4e-02**
	15	M1, M2	**4.0e-02**	6.8e-02	5.2e-01	**4.6e-02**	**3.9e-02**	**2.2e-03**	**2.2e-03**	**2.0e-04**	**1.0e-04**	**1.0e-04**	**1.0e-04**	**1.0e-04**
		M1, M3	**5.1e-03**	**1.0e-04**	6.3e-01	**9.2e-03**	**4.9e-03**	**2.2e-03**	**2.2e-03**	**4.0e-04**	**1.0e-04**	**1.0e-04**	**1.0e-04**	**1.0e-04**
		M2, M3	5.3e-02	**4.0e-04**	9.2e-01	8.6e-02	5.7e-02	**4.8e-02**	**4.8e-02**	**5.0e-02**	5.3e-02	9.2e-01	**4.8e-02**	**5.0e-02**
noise level σ	0 kb	M1, M2	4.5e-01	**3.2e-02**	6.3e-01	4.9e-01	4.7e-01	7.6e-02	7.6e-02	**1.3e-03**	**1.0e-04**	**1.0e-04**	**1.0e-04**	**1.0e-04**
		M1, M3	**7.8e-03**	**1.0e-04**	6.1e-01	**8.6e-03**	**6.8e-03**	**1.8e-03**	**1.8e-03**	**6.0e-04**	**1.0e-04**	**1.0e-04**	**1.0e-04**	**1.0e-04**
		M2, M3	**3.0e-02**	**1.0e-04**	8.8e-01	**4.2e-02**	**3.2e-02**	**4.5e-02**	**4.5e-02**	**2.6e-02**	**3.0e-02**	8.8e-01	**4.5e-02**	**2.6e-02**
	5 kb	M1, M2	**8.7e-03**	**2.5e-02**	8.0e-01	**3.6e-02**	**2.2e-02**	**5.0e-04**	**5.0e-04**	**2.0e-04**	**1.0e-04**	**1.0e-04**	**1.0e-04**	**1.0e-04**
		M1, M3	**4.0e-04**	**1.0e-04**	9.1e-01	**1.3e-03**	**5.0e-04**	**1.0e-04**	**1.0e-04**	**6.0e-04**	**1.0e-04**	**1.0e-04**	**1.0e-04**	**1.0e-04**
		M2, M3	**3.4e-02**	**1.0e-04**	9.1e-01	**4.8e-02**	**3.6e-02**	**5.0e-02**	**5.0e-02**	**3.7e-02**	**3.4e-02**	9.1e-01	**5.0e-02**	**3.7e-02**
	10 kb	M1, M2	**1.0e-04**	**3.0e-02**	7.6e-01	**1.0e-04**	**1.0e-04**	**1.0e-04**	**1.0e-04**	**1.0e-04**	**1.0e-04**	**1.0e-04**	**1.0e-04**	**1.0e-04**
		M1, M3	**1.0e-04**	**1.0e-04**	3.3e-01	**1.0e-04**	**1.0e-04**	**1.0e-04**	**1.0e-04**	**1.0e-04**	**1.0e-04**	**1.0e-04**	**1.0e-04**	**1.0e-04**
		M2, M3	**1.0e-02**	**1.0e-04**	5.5e-01	**1.2e-02**	**1.0e-02**	**7.9e-03**	**7.9e-03**	**7.1e-03**	**1.0e-02**	5.5e-01	**7.9e-03**	**7.1e-03**
	15 kb	M1, M2	**1.0e-04**	**3.5e-02**	6.6e-01	**1.0e-04**	**1.0e-04**	**1.0e-04**	**1.0e-04**	**2.0e-04**	**1.0e-04**	**1.0e-04**	**1.0e-04**	**1.0e-04**
		M1, M3	**1.0e-04**	**1.0e-04**	4.5e-01	**1.0e-04**	**1.0e-04**	**1.0e-04**	**1.0e-04**	**1.0e-04**	**1.0e-04**	**1.0e-04**	**1.0e-04**	**1.0e-04**
		M2, M3	**8.0e-04**	**1.0e-04**	6.4e-01	**2.0e-03**	**9.0e-04**	**9.0e-04**	**9.0e-04**	**5.0e-04**	**8.0e-04**	6.4e-01	**9.0e-04**	**5.0e-04**
resolution ρ	5 kb	M1, M2	7.8e-02	1.3e-01	1.2e-01	7.6e-02	7.8e-02	**3.8e-03**	**3.8e-03**	**3.8e-03**	**1.0e-04**	**1.0e-04**	**1.0e-04**	**1.0e-04**
		M1, M3	**1.4e-02**	**1.2e-02**	3.5e-01	**1.2e-02**	**1.2e-02**	**3.0e-02**	**3.0e-02**	6.2e-02	**1.0e-04**	**1.0e-04**	**1.0e-04**	**1.0e-04**
		M2, M3	4.7e-01	8.2e-02	**9.9e-03**	4.9e-01	4.7e-01	6.2e-01	6.2e-01	7.6e-01	4.7e-01	**9.9e-03**	6.2e-01	7.6e-01
	10 kb	M1, M2	**5.6e-03**	**1.9e-02**	7.4e-01	**2.7e-03**	**4.0e-03**	**1.1e-03**	**1.1e-03**	**1.5e-03**	**1.0e-04**	**1.0e-04**	**1.0e-04**	**1.0e-04**
		M1, M3	**1.0e-04**	**1.0e-04**	**1.9e-02**	**1.0e-04**	**1.0e-04**	**2.0e-04**	**2.0e-04**	**2.0e-04**	**1.0e-04**	**1.0e-04**	**1.0e-04**	**1.0e-04**
		M2, M3	**4.9e-02**	**1.4e-02**	**2.0e-02**	5.2e-02	**4.9e-02**	7.4e-02	7.4e-02	1.1e-01	**4.9e-02**	**2.0e-02**	7.4e-02	1.1e-01
	25 kb	M1, M2	**1.0e-04**	**1.0e-04**	6.3e-01	**1.0e-04**	**1.0e-04**	**1.0e-04**	**1.0e-04**	**1.0e-04**	**1.0e-04**	**1.0e-04**	**1.0e-04**	**1.0e-04**
		M1, M3	**1.0e-04**	**1.0e-04**	**1.0e-04**	**1.0e-04**	**1.0e-04**	**1.0e-04**	**1.0e-04**	**1.0e-04**	**1.0e-04**	**1.0e-04**	**1.0e-04**	**1.0e-04**
		M2, M3	**1.0e-04**	**1.0e-04**	**1.0e-04**	**1.0e-04**	**1.0e-04**	**1.0e-04**	**1.0e-04**	**1.0e-04**	**1.0e-04**	**1.0e-04**	**1.0e-04**	**1.0e-04**
	50 kb	M1, M2	**1.0e-04**	**1.0e-04**	**2.0e-04**	**1.0e-04**	**1.0e-04**	**1.0e-04**	**1.0e-04**	**1.0e-04**	**1.0e-04**	**1.0e-04**	**1.0e-04**	**1.0e-04**
		M1, M3	**1.0e-04**	**1.0e-04**	**2.0e-04**	**1.0e-04**	**1.0e-04**	**1.0e-04**	**1.0e-04**	**1.0e-04**	**1.0e-04**	**1.0e-04**	**1.0e-04**	**1.0e-04**
		M2, M3	**1.0e-04**	**1.0e-04**	**5.0e-04**	**1.0e-04**	**1.0e-04**	**1.0e-04**	**1.0e-04**	**1.0e-04**	**1.0e-04**	**5.0e-04**	**1.0e-04**	**1.0e-04**
global	-	M1, M2	**1.0e-04**	**1.0e-04**	1.9e-01	**1.0e-04**	**1.0e-04**	**1.0e-04**	**1.0e-04**	**1.0e-04**	**1.0e-04**	**1.0e-04**	**1.0e-04**	**1.0e-04**
		M1, M3	**1.0e-04**	**1.0e-04**	1.8e-01	**1.0e-04**	**1.0e-04**	**1.0e-04**	**1.0e-04**	**1.0e-04**	**1.0e-04**	**1.0e-04**	**1.0e-04**	**1.0e-04**
		M2, M3	**1.0e-04**	**1.0e-04**	6.6e-01	**1.0e-04**	**1.0e-04**	**1.0e-04**	**1.0e-04**	**1.0e-04**	**1.0e-04**	6.6e-01	**1.0e-04**	**1.0e-04**

cell line	accession number	measure	M1	M2	M3	M4
H1-hESC	4DNFI9GMP2J8	TPR	14.13	11.48	9.71	3.84
		TNR	93.90	97.77	97.97	99.24
		PPV	35.65	55.14	53.30	54.78
		bACC	54.02	54.62	53.84	51.54
		GM	36.42	33.50	30.85	19.52
		F1c	20.24	19.01	16.43	7.18
		FMc	22.44	25.16	22.76	14.50
		JI	11.26	10.50	8.95	3.72
		AHR	59.90	11.48	9.71	17.00
		FGC	79.08	54.92	53.65	99.12
		F1r	68.17	18.99	16.45	29.02
		FMr	68.83	25.11	22.83	41.05
		nAF	32,644	6,257	5,294	9,263
		nSP	26,210	11,765	12,018	3,864
	4DNFI6HDY7WZ	TPR	13.84	7.61	5.07	2.50
		TNR	92.19	98.31	98.99	99.40
		PPV	29.76	51.86	54.60	49.89
		bACC	53.01	52.96	52.03	50.95
		GM	35.72	27.35	22.41	15.77
		F1c	18.89	13.27	9.28	4.77
		FMc	20.29	19.87	16.64	11.17
		JI	10.43	7.11	4.87	2.44
		AHR	60.29	7.61	5.07	11.83
		FGC	71.89	51.48	54.60	98.11
		F1r	65.58	13.26	9.28	21.11
		FMr	65.84	19.79	16.64	34.07
		nAF	32,856	4,147	2,765	6,446
		nSP	31,496	8,415	5,989	2,757
GM12878	ENCFF993FGR	TPR	17.76	2.57	4.70	4.08
		TNR	96.33	97.74	96.56	99.33
		PPV	41.23	14.15	16.54	47.01
		bACC	57.05	50.15	50.63	51.71
		GM	41.37	15.84	21.30	20.14
		F1c	24.83	4.35	7.32	7.51
		FMc	27.06	6.03	8.82	13.85
		JI	14.18	2.22	3.80	3.90
		AHR	60.32	2.57	4.70	15.55
		FGC	82.70	14.17	16.56	97.54
		F1r	69.76	4.35	7.32	26.83
		FMr	70.63	6.03	8.82	38.95
		nAF	21,970	935	1,712	5,665
		nSP	18,127	6,762	14,076	3,173
	ENCFF216QQM	TPR	20.41	8.45	5.02	2.16
		TNR	86.91	98.80	99.40	99.67
		PPV	18.44	50.59	54.99	48.79
		bACC	53.66	53.62	52.21	50.92
		GM	42.11	28.89	22.34	14.68
		F1c	19.37	14.48	9.20	4.14
		FMc	19.40	20.67	16.62	10.27
		JI	10.73	7.80	4.82	2.11
		AHR	78.05	8.45	5.02	9.10
		FGC	51.73	50.41	55.23	97.96
		F1r	62.22	14.47	9.21	16.65
		FMr	63.54	20.63	16.65	29.85
		nAF	28,425	3,076	1,829	3,313
		nSP	51,099	6,539	3,547	1,621

Supplementary Table 7. Classification and recognition measures for each real Hi-C matrix – resolution set to 10 kb. The following encoding is adopted: M1 = StripePy, M2=Chromosight, M3=StripeCaller, M4=Stripenn.

tool	elapsed real time	max rss (Mb)	performance improcement
M1	1m 11.91s ± 0.18s	4109.95 ± 8.54	2.13x
M2	4m 17.4s ± 0.41s	2984.23 ± 30.85	7.61x
M3	33.82s ± 0.29s	634.69 ± 0.53	1.0x
M4	37m 13.89s ± 6.44s	1671.41 ± 12.33	66.06x

Supplementary Table 8. Elapsed times, maximum resident set size, and performance improvements for contact map with accession number ENCFF993FGR – resolution set to 10 kb. The following encoding is adopted: M1 = StripePy, M2=Chromosight, M3=StripeCaller, M4=Stripenn.
Timings were measured on a workstation equipped with an AMD Ryzen 9 7950X3D 16-Core Processor, 4x32GB of DDR5 4800 MT/s RAM, and running Fedora 41 (Linux 6.12.11). Temporary and output files were written to an NVMe drive (PCIE 4.0) with plenty of free space available. Values shown in the table correspond to the average and standard error of measurements across 5 independent runs for each tool.

Supplementary Table 9. Comparing normalizations. We report classification and recognition measures, number of anchors found (nAF), and number of stripes predicted (nSP) obtained when running StripePy on the real Hi-C contact maps with no (default), ice and scale normalizations. Here, resolution is set to 10 kb.

	GM12878						H1-hESC					
	4DNFI9GMP2J8			4DNFI6HDY7WZ			ENCFF993FGR			ENCFF216QQM		
measure	NONE	ICE	SCALE	NONE	ICE	SCALE	NONE	ICE	SCALE	NONE	ICE	SCALE
TPR	14.13	4.24	7.44	13.84	7.09	7.57	17.76	7.78	11.10	20.41	10.26	12.15
TNR	93.90	98.28	98.00	92.19	96.92	96.87	96.33	98.08	97.96	86.91	95.95	95.00
PPV	35.65	37.05	47.03	29.76	35.48	36.67	41.23	37.04	44.06	18.44	26.88	26.06
bACC	54.02	51.26	52.72	53.01	52.00	52.22	57.05	52.93	54.53	53.66	53.10	53.58
GM	36.42	20.42	27.01	35.72	26.20	27.09	41.37	27.62	32.97	42.11	31.37	33.97
F1c	20.24	7.61	12.85	18.89	11.81	12.55	24.83	12.86	17.73	19.37	14.85	16.57
FMc	22.44	12.54	18.71	20.29	15.86	16.66	27.06	16.97	22.11	19.40	16.60	17.80
JI	11.26	3.96	6.87	10.43	6.28	6.70	14.18	6.87	9.73	10.73	8.02	9.04
AHR	59.90	27.02	34.33	60.29	35.58	37.43	60.32	35.96	42.21	78.05	45.87	51.00
FGC	79.08	92.69	90.08	71.89	81.06	83.11	82.70	87.66	87.15	51.73	67.75	64.28
F1r	68.17	41.84	49.71	65.58	49.46	51.62	69.76	51.00	56.87	62.22	54.70	56.87
FMr	68.83	50.04	55.61	65.84	53.71	55.77	70.63	56.15	60.65	63.54	55.74	57.26
nAF	32,644	14,722	18,708	32,856	19,390	20,398	21,970	13,098	15,373	28,425	16,705	18,575
nSP	26,210	6,303	8,819	31,496	11,671	11,805	18,127	7,753	9,335	51,099	14,210	17,498
